# Evaluating the long‐term persistence of *Bacillus* spores on common surfaces

**DOI:** 10.1111/1751-7915.13267

**Published:** 2018-05-03

**Authors:** Kyle S. Enger, Jade Mitchell, Bharathi Murali, Dawn N. Birdsell, Paul Keim, Patrick L. Gurian, David M. Wagner

**Affiliations:** ^1^ Department of Biosystems & Agricultural Engineering Michigan State University East Lansing MI USA; ^2^ Department of Biological Sciences Northern Arizona University Flagstaff AZ USA; ^3^ Department of Civil, Architectural, and Environmental Engineering Drexel University Philadelphia PA USA; ^4^ Present address: Medical Advantage Group East Lansing MI USA

## Abstract

*Bacillus* spores resist inactivation, but the extent of their persistence on common surfaces is unclear. This work addresses knowledge gaps regarding biothreat agents in the environment to reduce uncertainty in risk assessment models. Studies were conducted to investigate the long‐term inactivation of *Bacillus anthracis* and three commonly used surrogate organisms – *B. cereus*,* B. atrophaeus* and *B. thuringiensis* on three materials: laminate countertop, stainless steel and polystyrene Petri dishes. Viable spores were measured at 1, 30, 90, 196, 304 and 1038 days. Twelve different persistence models were fit to the data using maximum likelihood estimation and compared. The study found that (1) spore inactivation was not log‐linear, as commonly modelled; (2) *B. thuringiensis* counts increased at 24 h on all materials, followed by a subsequent decline; (3) several experiments showed evidence of a ‘U’ shape, with spore counts apparently decreasing and then increasing between 1 and 304 days; (4) spores on polystyrene showed little inactivation; and (5) the maximum inactivation of 56% was observed for *B. atrophaeus* spores on steel at 196 days. Over the range of surfaces, time durations and conditions (humidity controlled vs. uncontrolled) examined, *B. thuringiensis* most closely matched the behaviour of *B. anthracis*.

## Introduction

*Bacillus anthracis* spores have been used as bioweapons since World War I (Christopher *et al*., 1997). It is important to consider the persistence of these spores in indoor environments after an attack, because spores can survive for decades. For example, Redmond *et al*. (1998) found viable *B. anthracis* spores in sugar samples used during World War I, even after 80 years of archiving. Aerosolized *B. anthracis* spores are capable of remaining airborne after an attack for up to 48 h, settling on surfaces based on physical properties such as ambient wind velocity, aerodynamic properties of the spores and hydrophobicity of the surface and possibly re‐aerosolizing (Sextro *et al*., 2002). Humans may be exposed to such spores via multiple exposure routes such as inhalation, cutaneous and ingestion. Credible and validated risk assessment for anthrax could aid in better management of bioterror incidents in the future. Because of numerous uncertainties about the persistence of spores, previous exposure assessments could not adequately consider the decay of spores over time (Canter, 2007; Hong *et al*., 2010). In these situations, extrapolation from short‐term experiments may be problematic depending on the decay model selected. Hence, an understanding of long‐term persistence will aid in decision‐making about risk mitigation strategies (Mitchell‐Blackwood *et al*., 2011; Hamilton *et al*., 2015).

The objective of this study was to quantitatively evaluate the inactivation of *Bacillus* spores – *B. anthracis* Sterne*, B. cereus, B. thuringiensis* and *B. atrophaeus –* from commonly found indoor fomites such as laminate, stainless steel and polystyrene over 1038 days and compare them by fitting 12 different inactivation models published in the literature. This study provides a more detailed understanding of the inactivation kinetics of *Bacillus* species overall under common indoor conditions and will facilitate the development of models to assess relative risks for *B. anthracis* over long periods of time (Hong *et al*., 2012). This information can be useful for understanding health or economic threats arising from *Bacillus* spores, which not only present bioterrorism hazards, but are ongoing food safety and agricultural hazards as well.

Due to the pathogenicity, ease of dissemination and subsequent contamination, the use of pathogenic *B. anthracis* spores is restricted to Biosafety Level (BSL) 3 laboratories. Therefore, non‐pathogenic surrogates for *B. anthracis* spores that mimic the behaviour of the species of interest are useful for experimental purposes. Based on previous theoretical studies, the following spores of *B. anthracis* surrogates were used for this work – *B. anthracis* Sterne (attenuated non‐pathogenic strain of *B. anthracis*)*, B. cereus* (common foodborne pathogen sharing morphological similarities with *B. anthracis*)*, B. thuringiensis* (commonly used to produce a toxin used as an insecticide that is morphologically and genetically similar to *B. anthracis*) and *B. atrophaeus* (most commonly used surrogate) (Carrera *et al*., 2007; Greenberg *et al*., 2010; Tufts *et al*., 2013). While the recovery of spores from surfaces has been studied (Hodges *et al*., 2010; Rose *et al*., 2011; Calfee *et al*., 2013), to date, no published studies have experimentally compared different surrogates of pathogenic *B. anthracis* spores under indoor conditions. Therefore, an objective of this work was to compare long‐term persistence models among the spores of *B. anthracis* and its surrogates, to aid in selection of a suitable surrogate for *B. anthracis* spores for the future.

Various mathematical models for microbial inactivation are available. Many of these models pertain to thermal inactivation. Although high‐temperature conditions are not common in an office or indoor building condition, the suitability of these inactivation models for describing attenuation in the environment should be tested. This study evaluated 12 models to provide a basis for future model development of persistence data under ambient conditions.

Typically, four types of survival curves have been observed for bacterial inactivation: linear; curves with shoulders; curves with tailing; and sigmoidal curves (Xiong *et al*., 1999). Models developed over the last three decades for microbial inactivation included the concepts of population dynamics of the spores and are empirical in nature (Rodriguez *et al*., 1992; van Boekel, 2002; Corradini and Peleg, 2003; Peleg, 2003; Corradini *et al*., 2007). Table 1 shows a list of persistence models for microbial inactivation along with their equations and references that give the basis of inactivation along with experimental factors such as concentration, temperature, relative humidity or time. All the models have three parameters or fewer.

**Table 1:** Persistence models

Name	Equation	Reference
Exponential	$\left(\frac{N_{t}}{N_{0}}\right)=\text{exp}(-k_{1}t)$	Chick (1908)
Logistic	$\left(\frac{N_{t}}{N_{0}}\right)=\frac{2}{1+\text{exp}(-k_{1}t)}$	Gonzalez (1995)
Fermi	$\left(\frac{N_{t}}{N_{0}}\right)=\frac{1}{1+\text{exp}(k_{1}\left(t-k_{2}\right))}$	Peleg (2006)
Weibull	$\left(\frac{N_{t}}{N0}\right)=10^{-({\left(t/k_{1}\right)}_{2}^{k})}$	Coroller *et al*. (2006), Mafart *et al*. (2002)
Gamma	$\left(\frac{N_{t}}{N_{0}}\right)=\text{exp}\{(t^{k_{2}-1}){\text{exp}}^{\left(-\frac{t}{k_{2}}\right)}\}$	R Core Team (2013)
Lognormal	$\left(\frac{N_{t}}{N_{0}}\right)=1-\{(\text{ln}\left(t\right)-k_{1})/k_{2}\}$	Aragao *et al*. (2007)
Juneja and Marks 1 (JM1)	$\left(\frac{N_{t}}{N_{0}}\right)=1-(1-\text{exp}\left(-k_{1}t\right){)}^{k_{2}}$	Juneja *et al*. (2003)
Juneja and Marks 2 (JM2)	$\left(\frac{N_{t}}{N_{0}}\right)=\frac{1}{1+\text{exp}(k_{1}+k_{2}\text{log}\left(t\right))}$	Juneja *et al*. (2006)
Gompertz (2 parameters)	$\left(\frac{N_{t}}{N_{0}}\right)=\text{exp}[-\frac{k_{1}}{k_{2}}\text{exp}((k_{2}t)-1)]$	Wu *et al*. (2004)
Gompertz (3 parameters)	$\left(\frac{N_{t}}{N_{0}}\right)=10^{k_{1}\exp[-\exp(\frac{-k_{2}\exp\left(1\right)\left(k_{3}-t\right)}{k_{1}}+1)]}$	Gil *et al*. (2011)
Broken‐line	$\left(\frac{N_{t}}{N_{0}}\right)=\text{exp}\left(-k_{1}t\right),t<k_{3}$	Carrera *et al*. (2007) and Rogers *et al*. (2011)
	$\left(\frac{N_{t}}{N_{0}}\right)=\text{exp}\left(-k_{1}t+k_{2}\left(t-k_{3}\right)\right),t\geq k_{3}$	
	Slope changes at *t* = *k*_3_	
Double exponential	$\left(\frac{N_{t}}{N_{0}}\right)=k_{3}\text{exp}\left(-k_{1}t\right)+\left(1-k_{3}\right)\text{exp}\left(-k_{2}t\right)$	Abraham *et al*. (1990)

*N*(0) denotes the number of organisms initially applied at time, *t *= 0. *N*(*t*) denotes the number of organisms remaining at time *t*. *k*_1_, *k*_2_, and *k*_3_ denote parameters. They have different interpretations depending on the model.

The classic one‐parameter inactivation model, the exponential model, treats microbial inactivation similar to a chemical reaction (Chick, 1908). Logistic (Gonzalez, 1995), and Fermi (Peleg, 2006) models were previously established to delineate the sigmoid‐shaped survival curve in microbiology. These models were used to fit the non‐logarithmic survival response variable over time, thereby accounting for non‐linearity in the curves such as tailing or delays in inactivation due to clumping of spores. However, the delay in inactivation due to clumping depends on the number of spores present in the clumps, which was not addressed in either of the two models. The subpopulations of spores of differing resistances are responsible for tails and shoulders in the survival curves and other deviations from log‐linearity. JM1 (Juneja *et al*., 2003) and JM2 (Juneja *et al*., 2006) models were developed originally for predictive modelling to estimate the consequences of food processing operations on the fate of foodborne pathogens. Gompertz models such as Gz2 (Wu *et al*., 2004) and Gz3 (Gil *et al*., 2011) were originally developed for modelling population dynamics. To account for the asymmetrical sigmoidal kinetics, Gompertz models were used in predicting microbial survival. The robustness of Gompertz models to predict microbial populations under different temperatures has made them versatile for modelling purposes. The broken‐line model (Carrera *et al*., 2007; Rogers *et al*., 2011) was used to account for abrupt changes in the non‐linearity in inactivation curve. Points of abrupt changes known as ‘break points’ were developed originally to provide a better understanding of the kinetic parameters at different points of departure in the inactivation curve. The double exponential model (Abraham *et al*., 1990) was used to take into account differences in spore resistances towards decay or inactivation among subpopulations. This model accounts for two different subpopulations that follow a first‐order exponential decay with varying resistances. The Weibull, lognormal and gamma distributions were also fitted to the experimental data, as they are commonly used in microbial survival or inactivation.

## Results and discussion

### Inactivation from 0 to 304 days

An ANOVA was conducted on the entire data set with alpha equivalent to 0.05. All factors – species, fomite, time and the two‐way and three‐way interactions between them – were statistically significant predictors of log reduction. The differences between species were significantly different except for *B. anthracis* and *B. atrophaeus* (*P* = 0.8976). Differences between fomites were significantly different except for steel and laminate (*P* = 0.4992). Differences between the time points are best described by the model results.

**Figure 1:**
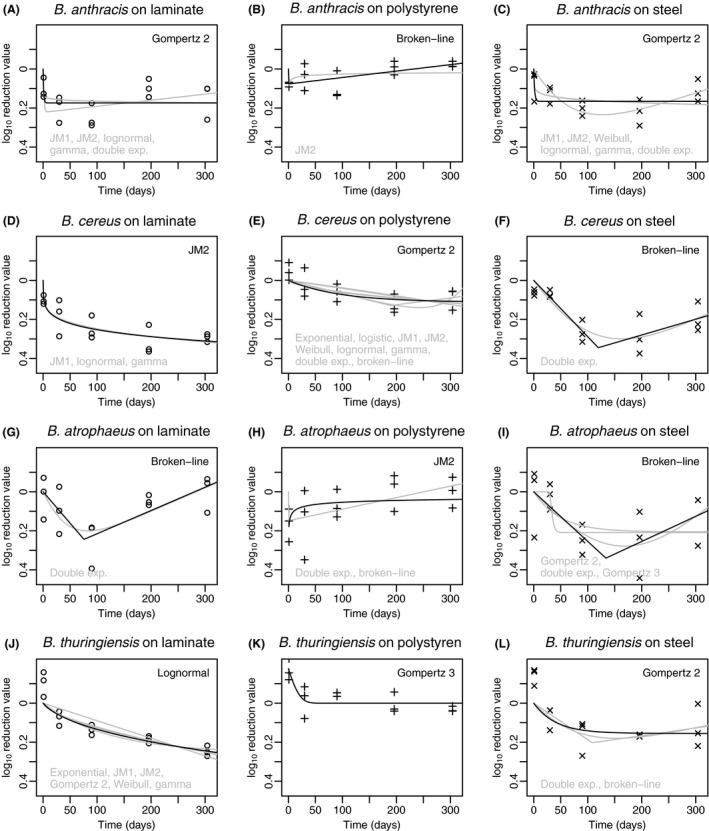
Best‐fit persistence models for the first five time points (0–304 days) shown in black. Models that were within 2 Bayesian Information Criterion units of the best‐fit model are shown in grey. Models for B. *anthracis* are shown in graphs A‐C; B. cereus in graphs D‐F; B. *atrophaeus* in graphs G‐I; and B. *thuringiensis* in graphs J‐L.

In general, there were < 0.5 log_10_ reductions in *Bacillus* spores on all fomites from 1 to 304 days (Fig. 1). No standard reduction trend in spores over time was observed for different species and fomites; however, there was a decrease followed by an increase that was observed in all species on steel fomites. Overall reduction on polystyrene for all the species was relatively lower than other fomites. This indicated that polystyrene might offer a better surface for spore protection leading to lower inactivation or that spores might adhere less strongly to polystyrene leading to higher recovery; however, it is not possible to distinguish the exact mechanism through this study. *Bacillus atrophaeus* had the least reduction over time on all three fomites. They are relatively smaller and morphologically different from *B. anthracis* Sterne, *B. thuringiensis* or *B. cereus* (Greenberg *et al*., 2010). This was previously found to result in lower adhesiveness of *B. atrophaeus* spores (Carrera *et al*., 2007).

### Inactivation from 0 to 1038 days

The results of an ANOVA for the 1038 time period were similar to the 304 daytime period above. All factors and interactions were statistically significant. The pairwise comparison between species resulted in significant differences except for *B. anthracis* and *B. atrophaeus* (*P* = 0.5424). There were also significant differences observed between steel and polystyrene as well as steel and laminate but not between polystyrene and laminate (*P* = 0.2335).

**Figure 2:**
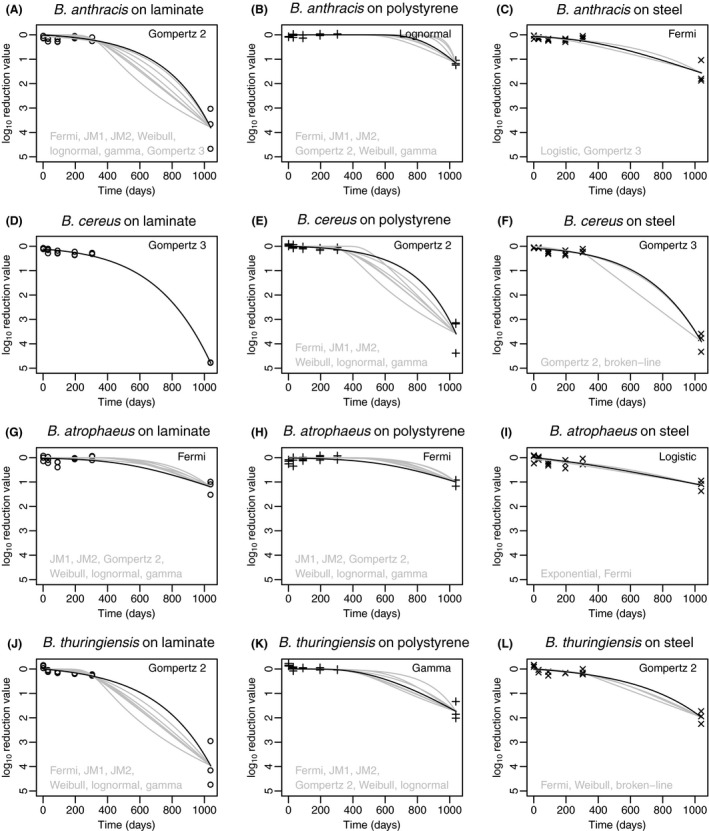
Best‐fit persistence models for the complete datasets (0–1038 days) shown in black. Models that were within 2 Bayesian Information Criterion units of the best‐fit model are shown in grey. Models for B. *anthracis* are shown in graphs A‐C; B. cereus in graphs D‐F; B. *atrophaeus* in graphs G‐I; and B. *thuringiensis* in graphs J‐L.

At 1038 days, log_10_ reductions ranging from 1 to 5 were observed, resulting in a concave‐down curve (Fig. 2). Again, after approximately 3 years of reduction, *B. atrophaeus* resulted in the lowest reduction ratio. On comparing across different fomites, reduction on polystyrene was the least, followed by stainless steel and then laminate. It is hence concluded from these results that spores are better inactivated on laminate and stainless steel surfaces or adhere more strongly to those surfaces. On comparing across the species and fomites together, it was observed that *B. anthracis* Sterne spores and *B. thuringiensis* spores behaved similarly on all three fomites, laminate, polystyrene and stainless steel. In Fig. 2, the steep declines seen for the last data point (i.e. the non‐humidity controlled observation) for *B. anthracis* are also observed for *B*. *cereus* and *B. thuringiensis*. However, *B. ce*reus shows a substantial decline on polystyrene and steel, whereas *B. thuringiensis* matches the more moderate decline of *B. anthracis*. Based on the indistinguishable difference between Bayesian information criterion (BIC) values, the Fermi model could be selected to describe inactivation of both *B. anthracis* and *B. thuringiensis* (Fig. 2).

### Comparison of two data sets

When comparing the best‐fit persistence models from Figs 1 and 2, 50% of the preferred models, that is models with BIC values within two units of the lowest BIC value obtained for all fomite–microbe data sets for 0–304 days, are also among the preferred models for 0–1038 days data sets. Furthermore, it is interesting that the double exponential model, which was among the preferred models for eight of 12 data sets from 0 to 304 days, was not among the preferred models for any of data sets in the time range 0–1038 days. Similarly, the Fermi model, which was not preferred for any of the data sets from 0 to 304 days, was preferred for 10 of 12 data sets in the time range 0–1038 days. The double exponential model indicates that subpopulations of spores with different inactivation kinetics could exist, whereas the Fermi model indicates the possibility of mixed population, presence of clumped spores or delayed inactivation (Peleg, 1996). Hence, the results are not completely conclusive with regard to best‐recommended models for long‐term persistence of all *Bacillus* spores; however, Table 2 lists best‐suited models for each data set in this work (that is those with BIC values within 2 of the lowest BIC obtained). Table 3 presents the models with the lowest BIC values summarized with their optimized parameter values.

**Table 2:** Summary of best‐fit persistence models with their BIC values

Time: 0–304 days		Time: 0–1038 days	
Model	BIC	Model	BIC
*Bacillus anthracis* on laminate			
JM1	−1.3395	Fermi	47.518
JM2	−1.3559	JM1	48.067
Gz2	−2.9085^a^	JM2	48.0588
Lognormal	−1.3641	Gz2	47.1561^a^
Gamma	−1.3277	Weibull	47.7211
Double exponential	−2.8372	Lognormal	48.0322
		Gamma	47.9527
		Gz3	48.8207
*Bacillus anthracis* on polystyrene			
JM2	−11.916	Fermi	−5.0897
Broken‐line	−13.426^a^	JM1	−5.0897
		JM2	−5.0894
		Gz2	−5.0897
		Weibull	−5.0852
		Lognormal	−5.0897^a^
		Gamma	−5.0897
*Bacillus anthracis* on steel			
JM1	−6.4031	Logistic	28.8845
JM2	−6.5533	Fermi	27.7263^a^
Gz2	−7.0459^a^	Gz3	29.4588
Weibull	−6.4817		
Lognormal	−6.6006		
Gamma	−6.4031		
Double exponential	−6.2113		
*Bacillus cereus* on laminate			
JM1	−12.093	Gz3	0.54715^a^
JM2	−12.194^a^		
Lognormal	−12.149		
Gamma	−12.093		
*Bacillus cereus* on polystyrene			
Exponential	−12.905	Fermi	39.0248
Logistic	−12.477	JM1	39.7709
JM1	−11.621	JM2	39.3491
JM2	−11.843	Gz2	38.7348^a^
Gz2	−12.949^a^	Weibull	39.0375
Weibull	−11.713	Lognormal	39.3461
Lognormal	−12.159	Gamma	39.2586
Gamma	−11.623		
Double exponential	−11.809		
Broken‐line	−11.81		
*Bacillus cereus* on steel			
Double exponential	−5.6618	Gz2	27.0329
Broken‐line	−6.5832^a^	Broken‐line	28.2564
		Gz3	26.5929^a^
*Bacillus atrophaeus* on laminate			
Double exponential	6.79885	Fermi	23.3633^a^
Broken‐line	5.39782^a^	JM1	24.8108
		JM2	24.8102
		Gz2	24.2735
		Weibull	24.8077
		Lognormal	24.8125
		Gamma	24.8109
*Bacillus atrophaeus* on polystyrene			
JM2	6.76612^a^	Fermi	16.6206^a^
Double exponential	7.57132	JM1	17.4876
Broken‐line	7.57132	JM2	17.4876
		Gz2	17.4639
		Weibull	17.4894
		Lognormal	17.4876
		Gamma	17.4876
*Bacillus atrophaeus* on steel			
Gz2	14.0205	Exponential	21.5841
Double exponential	13.4365	Logistic	20.967^a^
Broken‐line	13.0432^a^	Fermi	21.9064
Gz3	14.9432		
*Bacillus thuringiensis* on laminate			
Exponential	−8.4881	Fermi	48.1996
JM1	−8.7407	JM1	48.8759
JM2	−9.4054	JM2	48.9927
Gz2	−9.7387	Gz2	47.722^a^
Weibull	−9.0137	Weibull	48.1498
Lognormal	−10.085^a^	Lognormal	48.7965
Gamma	−8.7494	Gamma	48.5202
*Bacillus thuringiensis* on polystyrene			
Gz3	−11.968^a^	Fermi	20.1394
		JM1	20.0382
		JM2	20.0375
		Gz2	20.1024
		Weibull	20.0772
		Lognormal	20.038
		Gamma	20.0371^a^
*Bacillus thuringiensis* on steel			
Gz2	3.22912^a^	Fermi	18.5219
Double exponential	5.1404	Gz2	17.6264^a^
Broken‐line	5.09245	Weibull	19.3753
		Broken‐line	19.6094

All models shown have BIC values with two units of the lowest BIC value.

^a^.Lowest Bayesian information criterion (BIC).

**Table 3:** Summary of best‐fit models and their optimized parameters (95% confidence interval)

Time: 0–304 days				Time: 0–1038 days			
Model	*k*_1_	*k*_2_	*k*_3_	Model	*k*_1_	*k*_2_	*k*_3_
*Bacillus anthracis* on laminate							
Gz2	3.67E‐01 (2.07E‐01, 5.28E‐01)	−9.17E‐01 (−1.11E+00, −7.27E‐01)	NA	Gz2	9.16E‐04 (−6.75E‐02, 6.93E‐02)	3.37E‐03 (−2.81E‐02, 3.49E‐02)	NA
*Bacillus anthracis* on polystyrene							
Broken‐line	1.84E‐01 (−7.61E‐01, 1.13E+00)	1.85E‐01 (−7.61E‐01, 1.13E+00)	9.54E‐01 (−4.18E+00, 6.09E+00)	Lognormal	6.73E+00 (5.97E+00, 7.50E+00)	1.44E‐01 (−3.74E‐01, 6.62E‐01)	NA
*Bacillus anthracis* on steel							
Gz2	2.34E‐01 (8.21E‐02, 3.86E‐01)	−6.13E‐01 (−7.92E‐01, −4.35E‐01)	NA	Fermi	5.17E‐03 (−2.17E‐01, 2.27E‐01)	3.50E+02 (3.49E+02, 3.50E+02)	NA
*Bacillus cereus* on laminate							
JM2	1.41E+00 (−1.99E+00, −8.39E‐01)	2.55E‐01 (1.38E‐01, 3.73E‐01)	NA	Gz3	−1.93E+04 (−4.27E+04, 3.99E+03)	2.53E+00 (1.45E+00, 3.61E+00)	4.18E+03 (4.18E+03, 4.18E+03)
*Bacillus cereus* on polystyrene							
Gz2	2.71E‐03 (−6.57E‐02, 7.11E‐02)	−1.05E‐02 (−1.23E‐01, 1.02E‐01)	NA	Gz2	5.06E‐04 (−6.01E‐02, 6.12E‐02)	4.05E‐03 (−2.08E‐02, 2.89E‐02)	NA
*Bacillus cereus* on steel							
Broken‐line	6.65E‐03 (5.03E‐03, 8.26E‐03)	8.51E‐03 (5.93E‐03, 1.11E‐02)	1.19E+02 (1.19E+02, 1.20E+02)	Gz3	−4.44E+03 (−1.20E+04, 3.15E+03)	6.80E‐01 (−8.29E‐01, 2.19E+00)	3.32E+03 (3.32E+03, 3.32E+03)
*Bacillus atrophaeus* on laminate							
Broken‐line	7.42E‐03 (−1.95E‐04, 1.50E‐02)	1.02E‐02 (2.40E‐03, 1.79E‐02)	7.57E+01 (7.49E+01, 7.66E+01)	Fermi	5.30E‐03 (−3.87E‐01, 3.98E‐01)	5.31E+02 (5.30E+02, 5.31E+02)	NA
*Bacillus atrophaeus* on polystyrene							
JM2	−6.77E‐01 (−1.43E+00, 7.48E‐02)	−2.93E‐01 (−5.64E‐01, −2.16E‐02)	NA	Fermi	5.17E‐03 (−4.78E‐01, 4.89E‐01)	6.14E+02 (6.14E+02, 6.14E+02)	NA
*Bacillus atrophaeus* on steel							
Broken‐line	5.86E‐03 (2.75E‐03, 8.96E‐03)	8.82E‐03 (3.87E‐03, 1.38E‐02)	1.34E+02 (1.33E+02, 1.34E+02)	Logistic	3.13E‐03 (2.72E‐03, 3.55E‐03)	NA	NA
*Bacillus thuringiensis* on laminate							
Lognormal	6.14E+00 (5.59E+00, 6.68E+00)	2.45E+00 (1.36E+00, 3.54E+00)	NA	Gz2	1.36E‐03 (−5.92E‐02, 6.19E‐02)	2.91E‐03 (−2.81E‐02, 3.39E‐02)	NA
*Bacillus thuringiensis* on polystyrene							
Gz3	8.75E‐01 (−3.57E+02, 3.58E+02)	1.01E‐02 (−2.46E+02, 2.46E+02)	1.69E+01 (−2.97E+02, 3.31E+02)	Gamma	6.78E+01 (6.74E+01, 6.81E+01)	8.26E+00 (−5.70E+00, 2.22E+01)	NA
*Bacillus thuringiensis* on steel							
Gz2	8.76E‐03 (−8.41E‐02, 1.02E‐01)	−2.46E‐02 (−1.45E‐01, 9.59E‐02)	NA	Gz2	1.04E‐03 (−3.98E‐02, 4.19E‐02)	2.33E‐03 (−2.55E‐02, 3.02E‐02)	NA

It is unclear how much confidence to place in the data collected at 1038 days, because the fomites were subjected to drier conditions on average after the 304 daytime point. Drier conditions may have additionally stressed the spores (delaying germination) or reduced adhesion (increasing recovery). Nonetheless, the data suggest that spore inactivation or adhesion may accelerate over time or under reduced relative humidity due to pronounced concavity observed in all curves fitted across 0–1038 days. An alternate explanation is that the recovery percentage decreases as the number of spores decreases, which is suggested by the lower recoveries reported by CDC and discussed further in the next section of this study (Hodges *et al*., 2010; Rose *et al*., 2011). Despite these uncertainties, long‐term quantitative data on *Bacillus* spore persistence are scarce, and the results presented here may prove useful until additional multiyear persistence experiments can be conducted (Greenberg *et al*., 2010). The comparison and presentation of both findings (Figs 1 and 2) for the 304 day and 1038 day data sets are useful to illustrate the potential differences over a longer period of time and under different humidity conditions. For application of this work in risk assessment, it should also be noted that information on actual environmental conditions over many years is often unknown outside of a controlled environment and is a source of uncertainty, which cannot feasibly be reduced.

### Increased recoveries at 24 h time step

Recovery of spores 24 h after application was high, with median recoveries from 71% to 145% depending on the type of *Bacillus* species and the fomite used. In contrast to our finding, the US Centers for Disease Control and Prevention (CDC) documented lower recoveries in the range of 16–55% for the recovery of *B. anthracis* Sterne spores from stainless steel fomites, 2 h after application, using a swabbing technique similar to the one described here (Hodges *et al*., 2010). Another recovery study by the CDC reported about 30% recovery for *B. anthracis* Sterne spores from steel when recovered using sponges, 2 h after application (Rose *et al*., 2011). It is however not certain why the recovery in our study was so high compared to others, but it should be noted that, in this study, we applied ≥ 100 times as many spores (~ 2.5 × 10^6^) in order to allow measurement of high log_10_ reductions over much longer periods of time (Hodges *et al*., 2010; Rose *et al*., 2011). Additionally, if limited fissures or crevices on the surface of the fomites could effectively sequester spores, applying more spores would lead to higher recovery percentages, as a smaller fraction of the total number of spores would be shielded from the swabbing technique. Recovery of *Bacillus* spores has been shown to decrease with increasing surface roughness (Calfee *et al*., 2013). Over short durations, the recovery of *B. anthracis* Sterne spores was observed to significantly decrease, while the recovery of *B. thuringiensis* spores was consistently found to initially increase then decrease with time (Murali, 2014). Additional differences between the CDC methods and those used herein may have contributed to contrasting outcomes as well: (1) the swabs and the type of steel differed; and (2) we quantified spores immediately after swabbing at each time point, while in the CDC studies, the swabs were shipped overnight on cold packs to several different laboratories.

A two‐sided *t*‐test was used to determine whether the changes in concentrations at 24 h differed significantly from 0 (i.e. 100% recovery). Although several experiments showed significant differences initially, none remained significant after correcting for multiple comparisons. *Bacillus thuringiensis* on polystyrene and steel had a significant increase in detectable spores at 24 h. No other species approached a significant *increase* in spores at 24 h; however, after correction for multiple comparisons, these findings were not significant. The results indicated behaviour similar to an ‘activation shoulder’ 24 h after applying *B. thuringiensis* spores to all three fomites. There was an apparent (though non‐significant) increase in viable spores early in the persistence experiment, followed by a long decline. A similar increase followed by decrease in recovery of *B. thuringiensis* spores from porous fomite was observed, which was hypothesized and supported by evidence including SEM images to be caused by the combined effects of clumping, moisture content and the culturability of these spores (Murali, 2014). The exact basis of the significant increase in *B. thuringiensis* at 24 h is not known, but there are indications that the apparent ‘activation’ might occur due to multiple mechanisms; spores could actually reactivate from a dormant state, or there could be further dispersion of clumps of spores (Corradini and Peleg, 2003). In one study, temperatures of ~ 100°C are associated with an ‘activation shoulder’ lasting for minutes to hours for Bacillus spores (Corradini and Peleg, 2003). Nutrient‐induced germination consists of two processes including a lag time which varies from minutes to hours. *Bacillus subtilis* spores heat activated at 75°C demonstrated heterogeneity in germination due to stochastic gene expression (Zhang *et al*., 2013). It is established that germination rates of Bacillus spores are affected by temperatures with greater activation at higher temperatures (30–33°C) Granum *et al*., 2013, compared to lower temps (3–8°C) (Rose *et al*. 2011) The temperature parameters in our study did not exceed 37°C, which is suitable for germination; therefore, our ‘activation’ results suggest the existence of independent convergent mechanisms – reproduction and breaking up of clumps – that confer behaviours similar to an ‘activation shoulder’.

Growth can be excluded, as fomites were kept dry, and essentially no nutrient was available on the fomites; however, we did not evaluate growth explicitly. Solon *et al*. (2012) suspected that germination and multiplication may occur during extraction procedures, but the extraction procedure used here is brief.

### Apparent ‘U’ shape in the persistence curves

Upon visual inspection, some experiments appeared to show a ‘U‐ shaped pattern in persistence between 1 and 304 days (Fig. 1). A statistical test (Table 4) to detect this pattern indicated a significant ‘U’ shape for all four species on the steel fomite. After correcting for multiple comparisons, the ‘U’ shape remained significant only for *B. anthracis*, but *B. cereus* closely approached significance. It is unclear what might have caused the apparent decrease followed by an increase (‘U’ shape) in *Bacillus* spores on steel fomites (Fig. 1; Table 4). We speculate that spores were clumped after elution early in the experiments and became dispersed later, increasing the number of colony‐forming units detected (Murali & Mitchell, unpublished).

**Table 4:** Summary of statistical test results regarding ‘U’‐shaped curve

Species	Fomite	Test for ‘U’ shape^a^, *P*‐values	
		Uncorrected	Corrected
*Bacillus anthracis*	Laminate	0.304	0.920
*Bacillus anthracis*	Polystyrene	***	***
*Bacillus anthracis*	Steel	0.003**	0.031**
*Bacillus cereus*	Laminate	0.088*	0.494
*Bacillus cereus*	Polystyrene	0.075*	0.494
*Bacillus cereus*	Steel	0.005**	0.054*
*Bacillus atrophaeus*	Laminate	0.071*	0.494
*Bacillus atrophaeus*	Polystyrene	0.365	0.920
*Bacillus atrophaeus*	Steel	0.030**	0.238
*Bacillus thuringiensis*	Laminate	0.230	0.920
*Bacillus thuringiensis*	Polystyrene	0.375	0.920
*Bacillus thuringiensis*	Steel	0.014**	0.122

*P*‐values were corrected for multiple comparisons using Holm's method in the p.adjust() function in R 2.15.

^a^.Excluding the data collected at 1038 days.

* *P*‐value between 0.1 and 0.05.

** *P*‐value < 0.05.

*** *P*‐value not defined; this data set showed an apparent increase in spores from 1 to 304 days.

Although *Bacillus* spores are very durable, they were gradually inactivated or irreversibly bound to surfaces over the course of these experiments. They appear less durable on fomites than in long‐term experiments where spores were stored in phosphate‐buffered water (Evans & Curran, 1960); in that case, a variety of *Bacillus* and *Clostridium* spores generally showed < 1 log_10_ reduction after 3 years, although lowering the pH to 5 or briefly heating spores before storage could yield 4–6 log_10_ reductions after 2–3 years.

### Persistence models

For the 304 day data sets, the most successful models were as follows: double exponential (eight fits), broken‐line and JM2 (seven fits each), Gz2 (six fits) and gamma, JM1 and lognormal (five fits each). The results show that multiple models can often provide equivalent fits to the same data set. Furthermore, models with similar fits according to BIC often have dissimilar shapes (e.g. Figs 1i and 2e). A larger study with more frequent data collection (i.e. additional fomite samples) could have clarified model choice for these data sets. Only models having three or fewer parameters were tested (i.e. additional fomites), because there were only six time points at which *Bacillus* spores were measured. The applicable domain of these persistence models is for specific fomite–microbe combinations and is bounded by the range of time measured; therefore, extrapolation of the models beyond this time frame is not recommended. This study demonstrates the potential model uncertainty introduced when short‐term persistence models are used to predict long‐term survival. These models are often informative in integrated quantitative microbial risk assessment (QMRA) models for describing the attenuation of pathogens which may pose human health risks through environmental exposures (Haas *et al*., 2014).

We found evidence for a U shape (decreased recovery followed by increased recovery) among *Bacillus* species on steel. This indicates that strictly monotonic functions such as exponential or logistic models would not be ideal for *B. anthracis* spore persistence. Similar theory applies for any other one‐parameter model because such functions do not allow for a change in direction. Two‐ or three‐parameter models that were developed in more recent years take population dynamics into consideration, making such models more reliably predict inactivation of spores. The double exponential, broken‐line and JM2 models were the top three best‐fitting models of the twelve data sets. Other models such as JM1, JM2, gamma, Gz2 and Gz3 provided good fits consistently. Some models that appear to fit the data well may nonetheless have undesirable properties. The Fermi model and the Gz3 model do not necessarily equal 1 at time 0 (i.e. interpreted as < 100% of the spores being present at the beginning of the experiment); however, if the purpose of the analysis is to describe long‐term persistence, this may be a small disadvantage. The broken‐line model has an artificial shape and a poor theoretical justification regarding spore inactivation unless there is reason to believe that a brief event at a particular time altered the persistence of spores, giving rise to the ‘break’ point. (Muggeo, 2003). The exponential, Weibull, gamma, lognormal and Gz2 models are all directly obtained from the cumulative distribution functions of the probability distributions of the same name; these distributions are widely used in biology, engineering and other fields. Fitting one of these models to the data equates to the hypothesis that the persistence times of spores are described by the corresponding distribution (Peleg, 2003). More long‐term quantitative measurements of the persistence of *Bacillus* spores are needed to improve our understanding of long‐term risks associated with *B. anthracis* and other pathogenic spore‐forming bacteria. Although there are challenges with maintaining such experiments over several years, the necessary materials and techniques are relatively easy to employ. Additionally, recovery studies for spores should be assessed at different inoculum levels on the same fomites used in the persistence studies. Established microbiology laboratories can conduct these experiments, provided that careful documentation and training take place as laboratory staff turnover during multiyear periods may interfere with consistent methods. Although the efficiency of the recovery by wipe methods similar to the method used in this study has been well investigated by other researchers, it is variable as a function of initial inoculum dosage, surface type, humidity and time (GAO 2005, Brown *et al*., 2007; Estill *et al*., 2009; Rose *et al*., 2011; Krauter *et al*. 2012, Herzog *et al*. 2012). The recovery rates were also found to vary over the time period evaluated in the study. However, whether the organism is inactive or irreversibly bound, it would not pose a risk by most exposure pathways. Hence, the attenuation model is applicable to a QMRA even if we do not know the mechanism of attenuation.

## Experimental procedures

The experiments were conducted in the laboratory of David M. Wagner at Northern Arizona University. *Bacillus anthracis* (Sterne strain), *B. cereus* (unidentified strain), *B. atrophaeus* (Dugway strain) and *B. thuringiensis* (HD 1011 strain) spores were produced as follows. *Bacillus* vegetative cells were grown on sheep blood agar (SBA) plates (catalog # A10; Hardy Diagnostics, Santa Maria, CA, U.S.A.) at 37°C for 24 h. A single colony was resuspended in warmed phosphate‐buffered saline (PBS; 37°C, pH 7.0). That suspension (100 μL) was spread onto 2× Schaeffer's glucose agar (2× SG) plates (Nicholson and Setlow, 1990) and incubated at 37°C for 3 days. A loopful of culture was removed and suspended in 50 μL of sterile distilled water and examined with phase‐contrast microscopy. When over 90% of the cells had formed spores, the spore layer was removed using a cell scraper and resuspended by vortexing in 35 mL of cold (4°C) sterile distilled water. The spores were washed by shaking overnight at 4°C, followed by centrifugation at 2400 *g* for 30 min at 4°C. The supernatant was removed, and the pellet was resuspended in 35 mL cold (4°C) sterile distilled water. The washing and centrifugation process were repeated three or four times, until 95% of cells were spores by phase‐contrast microscopy.

Spores were applied to three types of fomites: (1) 2 × 2 cm pieces of laminate countertop (Frosty White #1573, matte finish; Wilsonart International, LLC, Temple, Texas, U.S.A.) sterilized by autoclaving at 121°C for 15 min, (2) stainless steel washers (Ace Hardware, U.S.A) washed in mild soap, rinsed four times in sterile water and autoclaved at 121°C for 15 min and (3) pieces of sterile polystyrene Petri dishes (Fisher Scientific, Hampton, NH) measuring 1–4 cm^2^. *Bacillus* spores were applied to the materials by placing a single 50 μL droplet of spore suspension containing spores in the range of 10^7^ CFU mL^−1^ in phosphate‐buffered saline (PBS; pH 7.0) in the centre of each fomite and allowing it to dry. Fomites were placed within covered sterile Petri dishes and stacked within an incubator in the dark at 26°C and 50–65% relative humidity, maintained by keeping an open tray of water in the incubator. The water dried out between 304 and 1038 days, meaning that the set of fomites assayed at 1038 days experienced drier conditions on average during that period. Humidity in the building is usually 20–40%, but during the ‘Arizona monsoon’ (early June to early September), it is 35–50% during the day and up to 85% at night. It was confirmed that the incubator used would have the same humidity as ambient humidity.

Spores were quantified at 1, 30, 90, 196, 304 and 1038 days (from June 2008 through March 2011), or six time points (*n* = 6) for each of the combinations of *Bacillus* species (*n* = 4) and fomite materials (*n* = 3). Three experimental trials (*n* = 3) were performed with duplicate samples at each time point (*n* = 2). Thus, a total of 432 fomites were used and the corresponding number of data points was collected for modelling (432 fomites = 6 time points × 4 species × 3 materials × 3 trials × 2 duplicates). Recovery of spores from fomites on day 0 (i.e. shortly after application to the fomite) was not attempted. Spores were quantified using a cotton swab wetted by PBS with 0.01% Tween‐80 (PBST) to completely swab the surface horizontally, vertically and diagonally, rotating the swab 120° when the direction was changed. Spores were eluted from the swab tip by placing it into 10 mL PBST and vortexing for 30–60 s. After serial dilutions were performed on the resulting suspension, the spot plate technique (Gaudy *et al*., 1963) was used to quantify spores. Briefly, each dilution level was quantified by placing three 20 μL droplets on a 15 × 100 mm SBA plate (cat. # A10; Hardy Diagnostics). Plates were incubated for 10–20 h at 37°C, and colonies were counted visually. The arithmetic mean of the three counts per fomite was used to estimate the number of spores remaining on each fomite. As experiments were begun in pairs using the same spore suspension, the results from each pair of experiments were also averaged, yielding three measurements for each of the six time points, for each of the 12 combinations of species and fomite materials.

This sampling method does not distinguish attenuation due to spore inactivation from attenuation due to irreversible binding to the fomite surface. To the extent that exposure pathways involve spores recoverable under these conditions, it may not be necessary to distinguish between inactivation and irreversible binding. However, scenarios, such as ingestion of fomites, which involve exposure to irreversibly bound spores, may require different models of attenuation that are specific to spore inactivation.

The models used in this study were as follows: exponential, logistic, Fermi, Juneja and Marks 1 (JM1; also known as log‐logistic model), Juneja and Marks 2 (JM2; also known as the two‐stage model), two‐parameter Gompertz (Gz2), three‐parameter Gompertz (Gz3), Weibull, lognormal, gamma, broken‐line (also known as biphasic exponential decay) and double exponential (Table 1). Persistence models were fitted to the data using maximum likelihood methods in R 2.15 (cran.r‐project.org). (The programming code is provided in Appendix S1). As the humidity conditions for the last set of fomites (assayed at 1038 days) were uncertain, models were fitted to two sets of data, (i) the first five time points, 0–304 days and (2) all six time points, 0–1038 days, to elucidate any differences. The raw data used in this study is provided in Table S1. The data were represented as log_10_ reductions from *N*_0_, the initial application concentration, and the errors were assumed to be normally distributed. The persistence models used in this study are presented in Table 1. Model comparison and selection were based on the BIC, which applies for both nested as well as non‐nested models. BIC is the ratio of the integrated likelihood of models of interest. Models differing from each other by ≤ 2 BIC units were considered equivalent (Bolker, 2008). Several of the data sets appeared to have a ‘U’ shape (decrease in spores followed by an apparent increase) between 1 day and 304 days; this was statistically tested *post hoc* using the ‘utest’ routine (Lind and Mehlum, 2010) with Stata 12.1. Unless otherwise noted, the threshold for statistical significance for all tests was α < 0.05. To correct for multiple comparisons, the Holm method (Holm, 1979) was employed in the p.adjust() function in R 2.15 (https://www.r-project.org/). This method controls the probability of Type I errors (false positives) when several hypotheses are evaluated.

## Conflict of interest

None declare.

## Supporting information


**Table S1**. Log reductions measured per species and fomite over time (days).

**Appendix S1**. Persistence Model Programming Code.
