# Supplementary material for: Evaluating the long‐term persistence of Bacillus spores on common surfaces
Source: Microb Biotechnol. 2018 May 3;11(6):1048–59. doi: 10.1111/1751-7915.13267 (PMC6196380; doi:10.1111/1751-7915.13267)
Supplement: Supplementary file 1 — Table S1. Log reductions measured per species and fomite over time (days). [file MBT2-11-1048-s001.docx]

Table S1. Log Reductions Measured per Species and Fomite over Time (days)

**Species Fomite Time (days) Log_10_ Reduction**

*B. anthracis* Laminate 1 0.089

*B. anthracis* Laminate 1 0.003

*B. anthracis* Laminate 1 0.178

*B. anthracis* Laminate 1 0.111

*B. anthracis* Laminate 1 0.144

*B. anthracis* Laminate 1 0.107

*B. cereus* Laminate 1 0.078

*B. cereus* Laminate 1 0.139

*B. cereus* Laminate 1 0.090

*B. cereus* Laminate 1 0.150

*B. cereus* Laminate 1 0.074

*B. cereus* Laminate 1 0.080

*B. atrophaeus* Laminate 1 -0.168

*B. atrophaeus* Laminate 1 0.056

*B. atrophaeus* Laminate 1 -0.032

*B. atrophaeus* Laminate 1 0.032

*B. atrophaeus* Laminate 1 0.162

*B. atrophaeus* Laminate 1 0.124

*B. thuringiensis* Laminate 1 0.008

*B. thuringiensis* Laminate 1 -0.070

*B. thuringiensis* Laminate 1 -0.147

*B. thuringiensis* Laminate 1 -0.084

*B. thuringiensis* Laminate 1 -0.275

*B. thuringiensis* Laminate 1 0.003

*B. anthracis* Laminate 30 0.156

*B. anthracis* Laminate 30 0.445

*B. anthracis* Laminate 30 0.139

*B. anthracis* Laminate 30 0.201

*B. anthracis* Laminate 30 0.136

*B. anthracis* Laminate 30 0.157

*B. cereus* Laminate 30 0.170

*B. cereus* Laminate 30 0.042

*B. cereus* Laminate 30 0.245

*B. cereus* Laminate 30 0.333

*B. cereus* Laminate 30 0.151

*B. cereus* Laminate 30 0.169

*B. atrophaeus* Laminate 30 0.052

*B. atrophaeus* Laminate 30 -0.089

*B. atrophaeus* Laminate 30 0.047

*B. atrophaeus* Laminate 30 0.149

*B. atrophaeus* Laminate 30 0.114

*B. atrophaeus* Laminate 30 0.353

*B. thuringiensis* Laminate 30 0.062

*B. thuringiensis* Laminate 30 0.021

*B. thuringiensis* Laminate 30 0.064

*B. thuringiensis* Laminate 30 0.071

*B. thuringiensis* Laminate 30 0.140

*B. thuringiensis* Laminate 30 0.096

*B. anthracis* Laminate 90 0.361

*B. anthracis* Laminate 90 0.202

*B. anthracis* Laminate 90 0.187

*B. anthracis* Laminate 90 0.164

*B. anthracis* Laminate 90 0.285

*B. anthracis* Laminate 90 0.291

*B. cereus* Laminate 90 0.302

*B. cereus* Laminate 90 0.083

*B. cereus* Laminate 90 0.303

*B. cereus* Laminate 90 0.280

*B. cereus* Laminate 90 0.250

*B. cereus* Laminate 90 0.297

*B. atrophaeus* Laminate 90 0.876

*B. atrophaeus* Laminate 90 0.172

*B. atrophaeus* Laminate 90 0.291

*B. atrophaeus* Laminate 90 0.095

*B. atrophaeus* Laminate 90 0.198

*B. atrophaeus* Laminate 90 0.177

*B. thuringiensis* Laminate 90 0.216

*B. thuringiensis* Laminate 90 0.116

*B. thuringiensis* Laminate 90 0.091

*B. thuringiensis* Laminate 90 0.132

*B. thuringiensis* Laminate 90 0.217

*B. thuringiensis* Laminate 90 0.068

*B. anthracis* Laminate 196 0.148

*B. anthracis* Laminate 196 0.060

*B. anthracis* Laminate 196 0.118

*B. anthracis* Laminate 196 0.169

*B. anthracis* Laminate 196 0.041

*B. anthracis* Laminate 196 0.059

*B. cereus* Laminate 196 0.353

*B. cereus* Laminate 196 0.376

*B. cereus* Laminate 196 0.275

*B. cereus* Laminate 196 0.186

*B. cereus* Laminate 196 0.366

*B. cereus* Laminate 196 0.344

*B. atrophaeus* Laminate 196 0.102

*B. atrophaeus* Laminate 196 0.011

*B. atrophaeus* Laminate 196 -0.010

*B. atrophaeus* Laminate 196 0.042

*B. atrophaeus* Laminate 196 0.052

*B. atrophaeus* Laminate 196 0.088

*B. thuringiensis* Laminate 196 0.127

*B. thuringiensis* Laminate 196 0.216

*B. thuringiensis* Laminate 196 0.136

*B. thuringiensis* Laminate 196 0.227

*B. thuringiensis* Laminate 196 0.201

*B. thuringiensis* Laminate 196 0.213

*B. anthracis* Laminate 304 0.274

*B. anthracis* Laminate 304 0.244

*B. anthracis* Laminate 304 0.139

*B. anthracis* Laminate 304 0.066

*B. anthracis* Laminate 304 0.127

*B. anthracis* Laminate 304 0.077

*B. cereus* Laminate 304 0.486

*B. cereus* Laminate 304 0.153

*B. cereus* Laminate 304 0.275

*B. cereus* Laminate 304 0.360

*B. cereus* Laminate 304 0.228

*B. cereus* Laminate 304 0.333

*B. atrophaeus* Laminate 304 -0.104

*B. atrophaeus* Laminate 304 -0.018

*B. atrophaeus* Laminate 304 0.052

*B. atrophaeus* Laminate 304 -0.126

*B. atrophaeus* Laminate 304 0.080

*B. atrophaeus* Laminate 304 0.137

*B. thuringiensis* Laminate 304 0.203

*B. thuringiensis* Laminate 304 0.229

*B. thuringiensis* Laminate 304 0.291

*B. thuringiensis* Laminate 304 0.255

*B. thuringiensis* Laminate 304 0.281

*B. thuringiensis* Laminate 304 0.226

*B. anthracis* Polystyrene 1 0.073

*B. anthracis* Polystyrene 1 0.063

*B. anthracis* Polystyrene 1 0.111

*B. anthracis* Polystyrene 1 0.074

*B. anthracis* Polystyrene 1 0.073

*B. anthracis* Polystyrene 1 0.073

*B. cereus* Polystyrene 1 -0.105

*B. cereus* Polystyrene 1 -0.078

*B. cereus* Polystyrene 1 -0.050

*B. cereus* Polystyrene 1 0.051

*B. cereus* Polystyrene 1 -0.035

*B. cereus* Polystyrene 1 -0.042

*B. atrophaeus* Polystyrene 1 0.106

*B. atrophaeus* Polystyrene 1 0.074

*B. atrophaeus* Polystyrene 1 -0.001

*B. atrophaeus* Polystyrene 1 0.379

*B. atrophaeus* Polystyrene 1 0.097

*B. atrophaeus* Polystyrene 1 0.510

*B. thuringiensis* Polystyrene 1 -0.254

*B. thuringiensis* Polystyrene 1 -0.201

*B. thuringiensis* Polystyrene 1 -0.114

*B. thuringiensis* Polystyrene 1 -0.192

*B. thuringiensis* Polystyrene 1 -0.122

*B. thuringiensis* Polystyrene 1 -0.116

*B. anthracis* Polystyrene 30 0.093

*B. anthracis* Polystyrene 30 0.129

*B. anthracis* Polystyrene 30 -0.002

*B. anthracis* Polystyrene 30 0.059

*B. anthracis* Polystyrene 30 -0.042

*B. anthracis* Polystyrene 30 -0.013

*B. cereus* Polystyrene 30 0.066

*B. cereus* Polystyrene 30 0.095

*B. cereus* Polystyrene 30 -0.069

*B. cereus* Polystyrene 30 -0.060

*B. cereus* Polystyrene 30 0.086

*B. cereus* Polystyrene 30 0.011

*B. atrophaeus* Polystyrene 30 0.094

*B. atrophaeus* Polystyrene 30 0.114

*B. atrophaeus* Polystyrene 30 -0.006

*B. atrophaeus* Polystyrene 30 -0.006

*B. atrophaeus* Polystyrene 30 0.262

*B. atrophaeus* Polystyrene 30 0.458

*B. thuringiensis* Polystyrene 30 -0.098

*B. thuringiensis* Polystyrene 30 -0.070

*B. thuringiensis* Polystyrene 30 -0.037

*B. thuringiensis* Polystyrene 30 -0.040

*B. thuringiensis* Polystyrene 30 0.068

*B. thuringiensis* Polystyrene 30 0.092

*B. anthracis* Polystyrene 90 0.185

*B. anthracis* Polystyrene 90 0.093

*B. anthracis* Polystyrene 90 0.206

*B. anthracis* Polystyrene 90 0.066

*B. anthracis* Polystyrene 90 0.015

*B. anthracis* Polystyrene 90 0.002

*B. cereus* Polystyrene 90 0.126

*B. cereus* Polystyrene 90 -0.004

*B. cereus* Polystyrene 90 0.112

*B. cereus* Polystyrene 90 0.105

*B. cereus* Polystyrene 90 0.065

*B. cereus* Polystyrene 90 -0.021

*B. atrophaeus* Polystyrene 90 0.045

*B. atrophaeus* Polystyrene 90 -0.062

*B. atrophaeus* Polystyrene 90 0.073

*B. atrophaeus* Polystyrene 90 0.189

*B. atrophaeus* Polystyrene 90 0.052

*B. atrophaeus* Polystyrene 90 0.124

*B. thuringiensis* Polystyrene 90 -0.038

*B. thuringiensis* Polystyrene 90 -0.070

*B. thuringiensis* Polystyrene 90 -0.087

*B. thuringiensis* Polystyrene 90 -0.016

*B. thuringiensis* Polystyrene 90 -0.015

*B. thuringiensis* Polystyrene 90 -0.053

*B. anthracis* Polystyrene 196 0.093

*B. anthracis* Polystyrene 196 -0.024

*B. anthracis* Polystyrene 196 -0.047

*B. anthracis* Polystyrene 196 -0.033

*B. anthracis* Polystyrene 196 -0.036

*B. anthracis* Polystyrene 196 0.015

*B. cereus* Polystyrene 196 0.047

*B. cereus* Polystyrene 196 0.095

*B. cereus* Polystyrene 196 0.285

*B. cereus* Polystyrene 196 0.067

*B. cereus* Polystyrene 196 0.180

*B. cereus* Polystyrene 196 0.114

*B. atrophaeus* Polystyrene 196 -0.045

*B. atrophaeus* Polystyrene 196 -0.033

*B. atrophaeus* Polystyrene 196 -0.119

*B. atrophaeus* Polystyrene 196 -0.044

*B. atrophaeus* Polystyrene 196 0.060

*B. atrophaeus* Polystyrene 196 0.147

*B. thuringiensis* Polystyrene 196 0.000

*B. thuringiensis* Polystyrene 196 0.062

*B. thuringiensis* Polystyrene 196 -0.060

*B. thuringiensis* Polystyrene 196 -0.054

*B. thuringiensis* Polystyrene 196 0.077

*B. thuringiensis* Polystyrene 196 0.008

*B. anthracis* Polystyrene 304 0.017

*B. anthracis* Polystyrene 304 -0.040

*B. anthracis* Polystyrene 304 -0.065

*B. anthracis* Polystyrene 304 -0.013

*B. anthracis* Polystyrene 304 -0.036

*B. anthracis* Polystyrene 304 0.009

*B. cereus* Polystyrene 304 0.031

*B. cereus* Polystyrene 304 0.080

*B. cereus* Polystyrene 304 0.134

*B. cereus* Polystyrene 304 0.171

*B. cereus* Polystyrene 304 0.114

*B. cereus* Polystyrene 304 0.008

*B. atrophaeus* Polystyrene 304 -0.073

*B. atrophaeus* Polystyrene 304 -0.073

*B. atrophaeus* Polystyrene 304 0.003

*B. atrophaeus* Polystyrene 304 -0.019

*B. atrophaeus* Polystyrene 304 0.114

*B. atrophaeus* Polystyrene 304 0.056

*B. thuringiensis* Polystyrene 304 0.008

*B. thuringiensis* Polystyrene 304 0.071

*B. thuringiensis* Polystyrene 304 0.019

*B. thuringiensis* Polystyrene 304 0.012

*B. thuringiensis* Polystyrene 304 0.011

*B. thuringiensis* Polystyrene 304 0.074

*B. anthracis* Stainless Steel 1 0.060

*B. anthracis* Stainless Steel 1 -0.003

*B. anthracis* Stainless Steel 1 0.023

*B. anthracis* Stainless Steel 1 0.046

*B. anthracis* Stainless Steel 1 0.103

*B. anthracis* Stainless Steel 1 0.240

*B. cereus* Stainless Steel 1 0.089

*B. cereus* Stainless Steel 1 0.034

*B. cereus* Stainless Steel 1 0.059

*B. cereus* Stainless Steel 1 0.032

*B. cereus* Stainless Steel 1 0.056

*B. cereus* Stainless Steel 1 0.101

*B. atrophaeus* Stainless Steel 1 -0.143

*B. atrophaeus* Stainless Steel 1 -0.033

*B. atrophaeus* Stainless Steel 1 -0.032

*B. atrophaeus* Stainless Steel 1 -0.087

*B. atrophaeus* Stainless Steel 1 0.316

*B. atrophaeus* Stainless Steel 1 0.167

*B. thuringiensis* Stainless Steel 1 -0.201

*B. thuringiensis* Stainless Steel 1 -0.134

*B. thuringiensis* Stainless Steel 1 -0.206

*B. thuringiensis* Stainless Steel 1 -0.112

*B. thuringiensis* Stainless Steel 1 -0.053

*B. thuringiensis* Stainless Steel 1 -0.120

*B. anthracis* Stainless Steel 30 0.220

*B. anthracis* Stainless Steel 30 0.140

*B. anthracis* Stainless Steel 30 0.088

*B. anthracis* Stainless Steel 30 0.103

*B. anthracis* Stainless Steel 30 0.144

*B. anthracis* Stainless Steel 30 0.073

*B. cereus* Stainless Steel 30 0.055

*B. cereus* Stainless Steel 30 0.042

*B. cereus* Stainless Steel 30 0.015

*B. cereus* Stainless Steel 30 0.168

*B. cereus* Stainless Steel 30 0.148

*B. cereus* Stainless Steel 30 0.024

*B. atrophaeus* Stainless Steel 30 0.011

*B. atrophaeus* Stainless Steel 30 -0.081

*B. atrophaeus* Stainless Steel 30 -0.010

*B. atrophaeus* Stainless Steel 30 0.032

*B. atrophaeus* Stainless Steel 30 0.068

*B. atrophaeus* Stainless Steel 30 0.119

*B. thuringiensis* Stainless Steel 30 0.080

*B. thuringiensis* Stainless Steel 30 -0.003

*B. thuringiensis* Stainless Steel 30 0.053

*B. thuringiensis* Stainless Steel 30 0.022

*B. thuringiensis* Stainless Steel 30 0.136

*B. thuringiensis* Stainless Steel 30 0.143

*B. anthracis* Stainless Steel 90 0.180

*B. anthracis* Stainless Steel 90 0.312

*B. anthracis* Stainless Steel 90 0.206

*B. anthracis* Stainless Steel 90 0.122

*B. anthracis* Stainless Steel 90 0.171

*B. anthracis* Stainless Steel 90 0.229

*B. cereus* Stainless Steel 90 0.160

*B. cereus* Stainless Steel 90 0.248

*B. cereus* Stainless Steel 90 0.294

*B. cereus* Stainless Steel 90 0.258

*B. cereus* Stainless Steel 90 0.307

*B. cereus* Stainless Steel 90 0.322

*B. atrophaeus* Stainless Steel 90 0.360

*B. atrophaeus* Stainless Steel 90 0.038

*B. atrophaeus* Stainless Steel 90 0.196

*B. atrophaeus* Stainless Steel 90 0.309

*B. atrophaeus* Stainless Steel 90 0.361

*B. atrophaeus* Stainless Steel 90 0.288

*B. thuringiensis* Stainless Steel 90 0.163

*B. thuringiensis* Stainless Steel 90 0.056

*B. thuringiensis* Stainless Steel 90 0.212

*B. thuringiensis* Stainless Steel 90 0.039

*B. thuringiensis* Stainless Steel 90 0.173

*B. thuringiensis* Stainless Steel 90 0.393

*B. anthracis* Stainless Steel 196 0.193

*B. anthracis* Stainless Steel 196 0.239

*B. anthracis* Stainless Steel 196 0.246

*B. anthracis* Stainless Steel 196 0.340

*B. anthracis* Stainless Steel 196 0.099

*B. anthracis* Stainless Steel 196 0.224

*B. cereus* Stainless Steel 196 0.381

*B. cereus* Stainless Steel 196 0.235

*B. cereus* Stainless Steel 196 0.394

*B. cereus* Stainless Steel 196 0.355

*B. cereus* Stainless Steel 196 0.158

*B. cereus* Stainless Steel 196 0.188

*B. atrophaeus* Stainless Steel 196 0.172

*B. atrophaeus* Stainless Steel 196 0.045

*B. atrophaeus* Stainless Steel 196 0.249

*B. atrophaeus* Stainless Steel 196 0.218

*B. atrophaeus* Stainless Steel 196 0.499

*B. atrophaeus* Stainless Steel 196 0.394

*B. thuringiensis* Stainless Steel 196 0.163

*B. thuringiensis* Stainless Steel 196 0.179

*B. thuringiensis* Stainless Steel 196 0.154

*B. thuringiensis* Stainless Steel 196 0.167

*B. thuringiensis* Stainless Steel 196 0.154

*B. thuringiensis* Stainless Steel 196 0.193

*B. anthracis* Stainless Steel 304 0.160

*B. anthracis* Stainless Steel 304 0.172

*B. anthracis* Stainless Steel 304 -0.004

*B. anthracis* Stainless Steel 304 0.115

*B. anthracis* Stainless Steel 304 0.059

*B. anthracis* Stainless Steel 304 0.204

*B. cereus* Stainless Steel 304 0.223

*B. cereus* Stainless Steel 304 0.288

*B. cereus* Stainless Steel 304 0.193

*B. cereus* Stainless Steel 304 0.258

*B. cereus* Stainless Steel 304 0.037

*B. cereus* Stainless Steel 304 0.196

*B. atrophaeus* Stainless Steel 304 0.014

*B. atrophaeus* Stainless Steel 304 0.074

*B. atrophaeus* Stainless Steel 304 -0.001

*B. atrophaeus* Stainless Steel 304 0.089

*B. atrophaeus* Stainless Steel 304 0.275

*B. atrophaeus* Stainless Steel 304 0.282

*B. thuringiensis* Stainless Steel 304 -0.005

*B. thuringiensis* Stainless Steel 304 0.011

*B. thuringiensis* Stainless Steel 304 0.255

*B. thuringiensis* Stainless Steel 304 0.187

*B. thuringiensis* Stainless Steel 304 0.217

*B. thuringiensis* Stainless Steel 304 0.099
