# Supplementary material for: Evaluating the long‐term persistence of Bacillus spores on common surfaces
Source: Microb Biotechnol. 2018 May 3;11(6):1048–59. doi: 10.1111/1751-7915.13267 (PMC6196380; doi:10.1111/1751-7915.13267)
Supplement: Supplementary file 2 — Appendix S1. Persistence Model Programming Code. [file MBT2-11-1048-s002.docx]

BaDataRead.r

#Reading in data

oldwd = getwd()

setwd('Directory’)

data = read.csv('File Name’,header=TRUE)

#data$SpeciesPlus = data$Species

data$Species = substr(data$Species,1,2)

data$Fomite = as.character(data$Fomite)

data$Fomite[data$Fomite=='filtered'] = 'Filtered'

data$Fomite[data$Fomite=='nonfiltered'] = 'Nonfiltered'

data$Experiment = mapply(paste,data$Species,data$Fomite)

data$Fomite = as.factor(data$Fomite)

data$Species = as.factor(data$Species)

print('These records had counts of 0:')

print(data[which(data$Count==0),])

table(data$Species,data$Fomite,data$Batch)

dLTm = aggregate(Count ~ Species + Batch + Fomite + log10 + Time + Conc, data=data, FUN=mean)

dLTmZeros = dLTm

dLTm$Count[which(dLTm$Count==0)] = 50

dLTm$pLeft = dLTm$Count / (dLTm$Conc)

dLTm$LR = -log10(dLTm$pLeft) #Calc. log10 reduction.

print('These aggregated records originally had counts of 0:')

print(dLTm[which(dLTmZeros$Count==0),])

nd = 0; datasets=NA #'datasets' is a convenient record of all the datasets generated

sdtp = NA #p-value for time trend in standard deviation, per dataset

plot(c(min(dLTm$Time),max(dLTm$Time)), c(0,2*sd(dLTm$LR)), pch='.',col='white',xlab='Intensity',ylab='SD',main='Time trends in SD for each dataset')

for (i in levels(dLTm$Species)){ #Splitting up into separate datasets per experimental group

for (j in levels(dLTm$Fomite)){

nd=nd+1

datasetName = datasets[nd] = paste(i,substr(j,1,1),"lt", sep='') #Generating & storing the dataset name

eval(parse(text=paste(datasetName,"= dLTm[which(dLTm$Species=='",i,"' & dLTm$Fomite=='",j,"'),]",sep=''))) #Creates the new sub-dataset

eval(parse(text=paste("write.csv(",datasetName,",'",datasetName,".csv')", sep=''))) #Writing a copy of the sub-dataset to .CSV (for U-test in Stata)

eval(parse(text=paste("SDs = aggregate(LR ~ Time, data=",datasetName,", FUN='sd')", sep=''))) #Regresses SDs on time

if (sum(is.na(SDs$LR)) > 0) SDs[which(is.na(SDs$LR)),'LR'] = 0 #If SD is undefined, set it to 0 (prevents errors from being thrown later).

eval(parse(text=paste(datasetName,"$expID='",datasetName,"'", sep=''))) #Stores the sub-dataset name in each of the sub-datasets

sdr = lm(LR ~ Time, data=SDs)

abline(coef(sdr)) #Plots the SD time trends

sdtp[nd] = summary(sdr)$coefficients['Time','Pr(>|t|)'] #Stores the SD time trend p-value

}

}

names(sdtp) = datasets

print('P values for a Time trend in standard deviations:')

print(format(sdtp,digits=2))

print("Corrected for multiple comparisons by Holm's method:")

print(format(p.adjust(sdtp,method='holm'),digits=2))

setwd(oldwd) #Resetting working directory to what it was before this code was run.

PersistFitKSVEv3.r

#This code requires datasets to have already been loaded into R, e.g., by BaDataRead.r

#V2 adds 3-parameter models and implements ability to select subsets of the available models for fitting.

#V3 does the following:

#renames the 1-param. 'general logistic' model to 'lg1' instead of 'gl'

#rolls the various model fits into a smaller group of functions (gutsMLEx)

#outputs results to their own folder

#saves brute-force estimates of ks into output

if (Sys.info()['sysname']=='Linux'){

setwd(dir)

}else{

setwd(dir)

}

###Setting options. See also initial parameter estimates near line 55.###

datafile = 'persistence modeling of bacteria,\nFormica fomite, swab, UV intensity' #A string which is used for a graph title.

dataset = 'lt' #The name of the dataset that you wish to analyze.

DesiredModels = c('default') #Use 'all' if you want to fit all available models. Use 'default' to fit a subset of decent models.

StartParamsFileType = 'NULL' #Can be 'output', 'start', or NULL. Defines the file from which starting values should be read.

StartParamsFileIndex = '2' #Index of desired file above.

if (!is.null(StartParamsFileType)){

ReadStartParams = read.csv(paste("Results/",dataset,"/",StartParamsFileType,dataset,StartParamsFileIndex,".csv", sep=''))

rownames(ReadStartParams) = ReadStartParams$X #Converting 1st column into row labels.

ReadStartParams = ReadStartParams[,-1] #Dropping 1st column.

}

#############

algorithm = 'Nelder-Mead' #Preferred optimization method for mle() to use. Could also try 'SANN', 'Nelder-Mead', 'CG', etc.

controllist=list(trace=0,maxit=40000,reltol=1e-8) ## ,reltol=1e-1); #Options for mle() for all models.

######

#if (!length(list.files(paste(getwd(),'/Results/',dataset,sep='')))){

print(paste('Creating directories for the output from the dataset',dataset,'...', sep=''))

if (!file.exists(paste('Results/',dataset, sep=''))) dir.create(paste('Results/',dataset, sep=''))

if (!file.exists(paste('Graphics/',dataset, sep=''))) dir.create(paste('Graphics/',dataset, sep=''))

#}

source('ParamTransformations.r') #Functions to transform parameters to facilitate optimization.

source('Transform.r') #Function for actually executing transformations or back-transformations.

source('PersistModelPredFuncs.r') #Reading in prediction functions. t is a vector of time values, ks is a matrix whose columns are parameters (k1, k2). If only 1 param., k1 is a vector of those.

source('PersistModelLikFuncs.r') #Reading in likelihood functions (which call the prediction functions above).

source('LikPlot1.r'); source('LikPlot2.r'); source('LikPlot3.r') #Functions for producing likelihood plots.

library(stats4); library(lattice)

eval(parse(text=paste("dat=",dataset,"[,c('uvint','LR')]",sep=''))) #A dataset produced by BaDataReadLongTerm.r is copied & named 'dat'.

#dat$Time = dat$Time / 24; #Changing time units from hours to days.

SDs = aggregate(LR ~ uvint, data=dat, FUN='sd') #Standard deviations of the data at each time point

SDs

SDs$LR[is.na(SDs$LR)] = 0

#Start with simple test for linear trend.

trend = lm(-LR ~ 0 + uvint, data=dat) #Forcing intercept through 0

summary(trend)

Slope = summary(trend)$coefficients[1,1]

pSlope = summary(trend)$coefficients[1,4]

dev.new()

plot(dat$uvint, -dat$LR, ylim=c(min(-dat$LR),max(max(-dat$LR),0)))

abline(0,Slope) #Might appear not to intercept at 0 because R automatically 'pads' the axes.

if (sign(Slope)==-1 & pSlope < 0.05){

title(main=paste('Test for trend, slope =',formatC(Slope,digits=2,format='E'),'p =',formatC(pSlope,digits=2,format='E'),'\nsignificant decline exists'))

}else{

title(main=paste('Test for trend, slope =',formatC(Slope,digits=2,format='E'),'p =',formatC(pSlope,digits=2,format='E'),'\nno significant decline exists'))

override = readline(paste("No significant decline over time by simple linear regression; hit 'y' to continue fitting: >"))

if (override != 'y') stop()

}

#Assigning Graph Symbols & param. transformations to the various available models, for a combination chart graphing many models at once.

#GSs = data.frame('model'=c('exponential','double expon.','expon. damped','biph. spline 2','biph. spline 3','Juneja & Marks 1','Juneja & Marks 2','logistic 1','logistic 2','Gompertz 2','Gompertz 3','Gomp.-Makeham','Weibull'),

# 'color'=c('blue','blue','blue','cyan','cyan','green','green','purple','purple','red','red','red','magenta'),

# 'lty'=c('solid','dashed','dotted','solid','dashed','solid','dashed','solid','dashed','solid','dashed','dotted','solid'))

GSs = data.frame('model'=c('exponential','logistic 1','logistic 2','expon. damped','Juneja & Marks 1','Juneja & Marks 2','Gompertz','Weibull','lognormal','gamma','biph. spline 2','biph. spline 3','double expon.','Gompertz 3','Gomp.-Makeham','sigmoid A','sigmoid B'),

'color'= c('blue', 'purple', 'purple', 'blue', 'green', 'green', 'red', 'black', 'brown', 'gray', 'cyan', 'cyan', 'blue', 'red', 'red', 'orange', 'orange'),

'lty' = c('solid', 'solid', 'dashed', 'dotted', 'solid', 'dashed', 'solid', 'solid', 'solid', 'solid', 'dashed', 'solid', 'dashed', 'dashed', 'dotted', 'solid', 'dashed'))

row.names(GSs) = c('ep','lg1','lg2','epd','jm1','jm2','gz','wb','ln','gam','bi','bi3','dep','gz3','gzm','sA','sB')

#Defining transformations. These MUST AGREE with the likelihood functions that end with '.TX'.

GSs[,c('k1','k2','k3','k4','k5')] = '-' #Making fields for transformation info

GSs[,'suffix'] = '' #Suffix for likelihood function, denoting transformation. Used later on in the gutsMLEx() functions.

#GSs['ep'] = ?

#GSs['lg1'] = ?

GSs['lg2',c('k1','k2','suffix')] = c('real2pos','real2pos','.T1')

#GSs['epd'] = ?

GSs['jm1',c('k1','k2','suffix')] = c('real2pos','real2pos','.T1')

#GSs['jm2'] = ?

GSs['gz',c('k1','k2','suffix')] = c('real2extreme','real2extreme','.T1')

GSs['wb',c('k1','suffix')] = c('real2extreme','.T1')

#GSs['ln'] = ?

GSs['gam',c('k1','suffix')] = c('real2extreme','.T1')

#GSs['bi'] = ?

GSs['bi3',c('k3','suffix')] = c('real2pos','.T1')

GSs['dep',c('k3','suffix')] = c('real2extreme','.T2')

GSs['gz3',c('k2','k3','suffix')] = c('real2pos','real2pos','.T1')

GSs['gzm',c('k1','k2','k3','suffix')] = c('real2extreme','real2extreme','real2extreme','.T1')

#GSs['sA'] = ?

#GSs['sB'] = ?

#########################

if (DesiredModels=='all'){DesiredModels = row.names(GSs)}

if (DesiredModels=='default'){DesiredModels = c('ep','lg1','lg2','jm1','jm2','gz','wb','ln','gam','dep','bi3','gz3')}

#DesiredModels = c('ep','dep','bi','bi3','epd','jm1','jm2','lg1','gz','wb') #The models to be fitted to the dataset (abbreviated). A subset of those in PersistModelLikFuncs.r.

start = data.frame('model'=rownames(GSs),'k1'=NA,'k2'=NA,'k3'=NA,'k4'=NA,'k5'=NA,'sd'=NA) #Starting values for parameters. These are always untransformed.

row.names(start) = start$model

start = start[,-1] #Removing the now-useless start$model column

start$sd = median(SDs$LR) #Populating the sd parameter that is common to all models, drawn from the original dataset

start$sd[start$sd==0] = 0.1 #Making a guess at sd, in the event that it's undefined (e.g., a single experiment is available).

if (!is.null(StartParamsFileType)){

start[rownames(ReadStartParams),c('k1','k2','k3','k4','k5','sd')] = ReadStartParams[,c('k1','k2','k3','k4','k5','sd')]

}

start$MLEmethod = algorithm #The optimization method chosen at the top of this code. Applied to all models.

start['wb','MLEmethod'] = 'SANN' #Overriding default method for Weibull model.

#If brand-new starting parameter values need to be used to override values from ReadStartParams, just set k1 to NA for the desired model.

#For example: start['gam','k1'] = NA

#Then the code below will substitute other parameter values for the desired model (gamma, in this example).

#####Inputting initial guesses of what the MLE parameter values should be. Can also override default optimization method.#####

if (is.na(start['ep','k1'])) start['ep','k1'] = 0.0002

if (is.na(start['lg1','k1'])) start['lg1','k1'] = 0.0001

if (is.na(start['lg2','k1'])) start['lg2',c('k1','k2')] = c(1e-4,1e4)

if (is.na(start['epd','k1'])) start['epd',c('k1','k2')] = c(1e-3,1e-3) #Gets screwy if k2 < 1e-3

if (is.na(start['jm1','k1'])) start['jm1',c('k1','k2')] = c(1e-5,1)

if (is.na(start['jm2','k1'])) start['jm2',c('k1','k2')] = c(-0.9,0.01)

if (is.na(start['gz','k1'])) start['gz',c('k1','k2')] = c(-1,-1e-1)

if (is.na(start['wb','k1'])) start['wb',c('k1','k2')] = c(1e6,0.05)

if (is.na(start['ln','k1'])) start['ln',c('k1','k2')] = c(10,5)

if (is.na(start['gam','k1'])) start['gam',c('k1','k2')] = c(1e5,0.1)

if (is.na(start['bi','k1'])) start['bi',c('k1','k2')] = c(0.007,0.007)

if (is.na(start['bi3','k1'])) start['bi3',c('k1','k2','k3')] = c(1e-4,1e-4,96); #startT['bi3','k3'] = 'real2pos'

if (is.na(start['dep','k1'])) start['dep',c('k1','k2','k3')] = c(1e-3,1e-5,1.5); #startT['dep','k3'] = 'real2prob' #NOTE: startT must agree with the transformations used in the nll.XXX.T() functions.

if (is.na(start['gz3','k1'])) start['gz3',c('k1','k2','k3')] = c(-0.1,1e-4,1e1)

if (is.na(start['gzm','k1'])) start['gzm',c('k1','k2','k3')] = c(1e-4,-1e-13,2e-8)

if (is.na(start['sA','k1'])) start['sA',c('k1','k2','k3')] = c(-1e3,0.0001,500)

if (is.na(start['sB','k1'])) start['sB',c('k1','k2','k3')] = c(0.1,1e6,2)

#####End starting parameters.#####

start = start[DesiredModels,] #Dumping starting values that will not be used.

start$n = nrow(dat)

startT = Transform(start,GSs,1)

outputT = startT[DesiredModels,] #Stores outputted parameter values & BICs

outputT$nll = NA

outputT$BIC = NA

outputT$kSource = 'start' #Flags k parameters as being the starting estimates. If mle() is successful, this will be flipped to 'mle' later.

#BICs = data.frame('ep'=NA,'bi'=NA,'epd'=NA,'jm1'=NA,'jm2'=NA,'lg1'=NA,'gz'=NA) #Vector of BICs that will be taken from 'output' above (purely for convenience)

##########################################################################

# Define decay functions

# ep ---> exponential

# dep ---> double exponential

# bi ---> biphasic spline with predefined breakpoint (defaults to 72)

# bi3 ---> biphasic spline with explicit 3rd parameter for breakpoint

# epd ---> exponential damped

# jm1 ---> Juneja&Marks(1)

# jm2 ---> Juneja&Marks(2)

# lg1 ---> 1 parameter logistic

# gz ---> Gompertz

##########################################################################

#####Fitting the models#####

#NOTE: If mle() succeeds, it yields an sd estimate, which is then reused by the brute force method.

#If the brute force method yields a lower BIC than mle(), the brute force k parameter estimates are kept, along with the mle() estimate of sd.

#However, if mle() fails, the brute force method uses the starting estimate for sd.

#Checking existence of output files, so as to avoid overwriting.

OutputFileIndex = 1

while(file.exists(paste("Results/",dataset,"/start",dataset,OutputFileIndex,".csv", sep=''))){

OutputFileIndex = OutputFileIndex + 1

}

saveMLE = function(DM,dataset,results,OutputFileIndex){ #Called by the gutsMLEx() functions below in order to save the full MLE object to disk.

save(results,file=paste("Results/",dataset,"/",DM,OutputFileIndex,".mle",sep=''),ascii=TRUE)

}

gutsMLE1 = function(DM,outputT,GSs,k1lim,k1sc){ #DM means 'desired model'. TODO: Get CIs around parameters. Something like sqrt(diag(solve(results@details$hessian)) to get std. err.

print(paste("=== Starting fit for",DM,"==="))

eval(parse(text=paste("kStart = as.list(startT['",DM,"', !is.na(startT['",DM,"',])])", sep=''))) #Making a list of the starting values for the k parameters & sd, to feed to mle().

kStart$MLEmethod = NULL #Dropping the MLE optimization method since it is not a parameter

kStart$n = NULL #Likewise dropping 'n' since it is not a parameter

print(kStart)

tryCatch(eval(parse(text=paste("results = mle(nll.",DM,GSs[DM,'suffix'],", start=kStart, method='",startT[DM,'MLEmethod'],"', control=controllist, fixed=list(data=dat))", sep='') )), error=function(err){print(paste('mle() failure, running brute force:',err))})

if (exists('results')){

print('Writing mle() results to outputT.')

outputT[DM,'kSource'] = 'mle'

j<-coef(results)

outputT[DM,'k1'] = j["k1"]

outputT[DM,'sd'] = j["sd"]

outputT[DM,'MLEmethod'] = results@call$method

outputT[DM,'nll'] = -logLik(results)

outputT[DM,'BIC'] = AIC(results,k=log(NROW(dat))) #BIC.

print("mle() results (ks are transformed, if applicable):")

print(outputT[DM,])

saveMLE(DM,dataset,results,OutputFileIndex)

}

output = Transform(outputT,GSs,0) #Need untransformed parameters for the likelihood plots.

gMLEs = LikPlot1(DM,k1lim,k1sc,output,dataset) #Now plotting the neg. log likelihood using brute force for different values of k1 to check the optimization above.

gMLEs$BIC = 2*gMLEs[,'nll'] + ncol(gMLEs)*log(nrow(dat)) #BIC based on the total number of k parameters, plus the sd parameter.

print("Brute force results (ks are untransformed):")

print(gMLEs)

#print(str(gMLEs))

nllR = gMLEs[,'nll'] / outputT[DM,'nll']

if (exists('results') & output[DM,'kSource']=='mle' & (nllR > 1.01 | nllR < 0.99)){warning("Negative log likelihoods from mle() and brute force differ by >1%!")}

if (!exists('results') || min(gMLEs[,'BIC']) < output[DM,'BIC']){

print('Writing brute force results to output; either mle() threw an error, or brute force BIC < mle BIC.')

output[DM,'k1'] = gMLEs['k1']

output[DM,'sd'] = gMLEs[1,'sd']

output[DM,'nll'] = gMLEs[1,'nll']

output[DM,'BIC'] = min(gMLEs$BIC)

output[DM,'kSource'] = 'BF' #Marking brute-force-derived estimates.

if (exists('results')){output[DM,'kSource'] = 'mBF'} #Marking brute-force-derived estimates following a 'successful' mle() fit.

outputT = Transform(output,GSs,1)

}

print(paste("=== Finished fit for",DM,"===")); print('')

return(outputT)

}

gutsMLE2 = function(DM,outputT,GSs,k1lim,k1sc,k2lim,k2sc){ #DM means 'desired model'.

print(paste("=== Starting fit for",DM,"==="))

eval(parse(text=paste("kStart = as.list(startT['",DM,"', !is.na(startT['",DM,"',])])", sep=''))) #Making a list of the starting values for the k parameters & sd, to feed to mle().

kStart$MLEmethod = NULL #Dropping the MLE optimization method since it is not a parameter

kStart$n = NULL #Likewise dropping 'n' since it is not a parameter

tryCatch(eval(parse(text=paste("results = mle(nll.",DM,GSs[DM,'suffix'],", start=kStart, method='",startT[DM,'MLEmethod'],"', control=controllist, fixed=list(data=dat))", sep='') )), error=function(err){print(paste('mle() failure, running brute force:',err))})

if (exists('results')){

print('Writing mle() results to outputT.')

outputT[DM,'kSource'] = 'mle'

j<-coef(results)

outputT[DM,'k1'] = j["k1"]

outputT[DM,'k2'] = j["k2"]

outputT[DM,'sd'] = j["sd"]

outputT[DM,'MLEmethod'] = results@call$method

outputT[DM,'nll'] = -logLik(results)

outputT[DM,'BIC'] = AIC(results,k=log(NROW(dat))) #BIC.

print("mle() results (ks are transformed, if applicable):")

print(outputT[DM,])

saveMLE(DM,dataset,results,OutputFileIndex)

}

output = Transform(outputT,GSs,0) #Need untransformed parameters for the likelihood plots.

gMLEs = LikPlot2(DM,k1lim,k1sc,k2lim,k2sc,output,dataset) #Now plotting the neg. log likelihood using brute force for different values of ks to check the optimization above.

gMLEs$BIC = 2*gMLEs[,'nll'] + ncol(gMLEs)*log(nrow(dat)) #BIC based on the total number of k parameters, plus the sd parameter.

print("Brute force results (ks are untransformed):")

print(gMLEs)

nllR = gMLEs[,'nll'] / outputT[DM,'nll']

if (exists('results') & outputT[DM,'kSource']=='mle' & (nllR > 1.01 | nllR < 0.99)){warning("Negative log likelihoods from mle() and brute force differ by >1%!")}

if (!exists('results') || min(gMLEs[,'BIC']) < output[DM,'BIC']){

print('Writing brute force results to output; either mle() threw an error, or brute force BIC < mle BIC.')

output[DM,'k1'] = gMLEs[1]

output[DM,'k2'] = gMLEs[2]

output[DM,'sd'] = gMLEs[1,'sd']

output[DM,'nll'] = gMLEs[1,'nll']

output[DM,'BIC'] = min(gMLEs$BIC)

output[DM,'kSource'] = 'BF' #Marking brute-force-derived estimates.

if (exists('results')){output[DM,'kSource'] = 'mBF'} #Marking brute-force-derived estimates following a 'successful' mle() fit.

outputT = Transform(output,GSs,1)

}

print(paste("=== Finished fit for",DM,"===")); print('')

return(outputT)

}

gutsMLE3 = function(DM,outputT,GSs,k1lim,k1sc,k2lim,k2sc,k3lim){ #DM means 'desired model'.

print(paste("=== Starting fit for",DM,"==="))

eval(parse(text=paste("kStart = as.list(startT['",DM,"', !is.na(startT['",DM,"',])])", sep=''))) #Making a list of the starting values for the k parameters & sd, to feed to mle().

kStart$MLEmethod = NULL #Dropping the MLE optimization method since it is not a parameter

kStart$n = NULL #Likewise dropping 'n' since it is not a parameter

tryCatch(eval(parse(text=paste("results = mle(nll.",DM,GSs[DM,'suffix'],", start=kStart, method='",startT[DM,'MLEmethod'],"', control=controllist, fixed=list(data=dat))", sep='') )), error=function(err){print(paste('mle() failure, running brute force:',err))})

if (exists('results')){

print('Writing mle() results to outputT.')

outputT[DM,'kSource'] = 'mle'

j<-coef(results)

outputT[DM,'k1'] = j["k1"]

outputT[DM,'k2'] = j["k2"]

outputT[DM,'k3'] = j["k3"]

outputT[DM,'sd'] = j["sd"]

outputT[DM,'MLEmethod'] = results@call$method

outputT[DM,'nll'] = -logLik(results)

outputT[DM,'BIC'] = AIC(results,k=log(NROW(dat))) #BIC.

print("mle() results (ks are transformed, if applicable):")

print(outputT[DM,])

saveMLE(DM,dataset,results,OutputFileIndex)

}

output = Transform(outputT,GSs,0) #Need untransformed parameters for the likelihood plots.

gMLEs = LikPlot3(DM,k1lim,k1sc,k2lim,k2sc,k3lim,output,dataset) #Now plotting the neg. log likelihood using brute force for different values of ks to check the optimization above.

gMLEs$BIC = 2*gMLEs[,'nll'] + ncol(gMLEs)*log(nrow(dat)) #BIC based on the total number of k parameters, plus the sd parameter.

print("Brute force results (ks are untransformed):")

print(gMLEs)

nllR = gMLEs[,'nll'] / outputT[DM,'nll']

if (exists('results') & outputT[DM,'kSource']=='mle' & (nllR > 1.01 | nllR < 0.99)){warning("Negative log likelihoods from mle() and brute force differ by >1%!")}

if (!exists('results') || min(gMLEs[,'BIC']) < output[DM,'BIC']){

print('Writing brute force results to output; either mle() threw an error, or brute force BIC < mle BIC.')

output[DM,'k1'] = gMLEs[,'k1']

output[DM,'k2'] = gMLEs[,'k2']

output[DM,'k3'] = gMLEs[,'k3']

output[DM,'nll'] = gMLEs[,'nll']

output[DM,'BIC'] = min(gMLEs$BIC)

output[DM,'kSource'] = 'BF' #Marking brute-force-derived estimates.

if (exists('results')){output[DM,'kSource'] = 'mBF'} #Marking brute-force-derived estimates following a 'successful' mle() fit.

outputT = Transform(output,GSs,1)

}

print(paste("=== Finished fit for",DM,"===")); print('')

return(outputT)

}

#Actually running the fits.

#row.names(GSs) = c('ep','lg1','lg2','epd','jm1','jm2','gz','wb','ln','gam','bi','bi3','dep','gz3','gzm','sA','sB')

if (sum(DesiredModels=='ep') >= 1){

outputT = gutsMLE1('ep',outputT,GSs,c(0.1,10),'*')}

if (sum(DesiredModels=='lg1') >= 1){

outputT = gutsMLE1('lg1',outputT,GSs,c(0.1,10),'*')}

if (sum(DesiredModels=='lg2') >= 1){

outputT = gutsMLE2('lg2',outputT,GSs,c(0.1,10),'*',c(0.1,10),'*')}

if (sum(DesiredModels=='epd') >= 1){

outputT = gutsMLE2('epd',outputT,GSs,c(0.1,10),'*',c(0.1,10),'*')}

if (sum(DesiredModels=='jm1') >= 1){

outputT = gutsMLE2('jm1',outputT,GSs,c(0.1,10),'*',c(.1,10),'*')}

if (sum(DesiredModels=='jm2') >= 1){

outputT = gutsMLE2('jm2',outputT,GSs,c(0.01,100),'*',c(0.01,3),'*')}

if (sum(DesiredModels=='gz') >= 1){

outputT = gutsMLE2('gz',outputT,GSs,c(.01,100),'*',c(.01,100),'*')}

if (sum(DesiredModels=='wb') >= 1){

outputT = gutsMLE2('wb',outputT,GSs,c(0.0001,10000),'*',c(0.01,100),'*')}

if (sum(DesiredModels=='ln') >= 1){

outputT = gutsMLE2('ln',outputT,GSs,c(0.01,100),'*',c(0.01,100),'*')}

if (sum(DesiredModels=='gam') >= 1){

outputT = gutsMLE2('gam',outputT,GSs,c(0.01,100),'*',c(0.01,100),'*')}

if (sum(DesiredModels=='bi') >= 1){

outputT = gutsMLE2('bi',outputT,GSs,c(0.01,100),'*',c(0.01,100),'*')}

if (sum(DesiredModels=='bi3') >= 1){

outputT = gutsMLE3('bi3',outputT,GSs,c(0.1,3),'*',c(0.1,3),'*',c(0,max(dat$uvint)))}

if (sum(DesiredModels=='dep') >= 1){

outputT = gutsMLE3('dep',outputT,GSs,c(0.01,100),'*',c(0.01,100),'*',c(0,3))}

if (sum(DesiredModels=='gz3') >= 1){

outputT = gutsMLE3('gz3',outputT,GSs,c(0.01,100),'*',c(0.01,100),'*',c(0,max(dat$uvint)))}

if (sum(DesiredModels=='gzm') >= 1){

outputT = gutsMLE3('gzm',outputT,GSs,c(0.01,100),'*',c(0.01,100),'*',c(-1e-3,1e-3))}

if (sum(DesiredModels=='sA') >= 1){

outputT = gutsMLE3('sA',outputT,GSs,c(-500,1000),'+',c(-500,1000),'+',c(-100,1000))}

if (sum(DesiredModels=='sB') >= 1){

outputT = gutsMLE3('sB',outputT,GSs,c(0.001,1000),'*',c(0.001,1000),'*',c(-10,10))}

#Back-transforming output MLEs of k-parameters that were obtained through transformation.

output = Transform(outputT,GSs,0)

#Adding value at time 0 returned by each model. Ideally would always be 1 (100% of spores at time 0).

output$p0 = NA

for (i in rownames(output)){

if (is.na(output[i,'k2'])){ #1-parameter models

eval(parse(text=paste("output['",i,"','p0'] = pred_",i,"(output['",i,"','k1'],0,FALSE)", sep='')))

} else if (is.na(output[i,'k3'])){ #2-parameter models

eval(parse(text=paste("output['",i,"','p0'] = pred_",i,"(output['",i,"','k1'],output['",i,"','k2'],0,FALSE)", sep='')))

} else { #3-parameter models

eval(parse(text=paste("output['",i,"','p0'] = pred_",i,"(output['",i,"','k1'],output['",i,"','k2'],output['",i,"','k3'],0,FALSE)", sep='')))

}

}

#dev.new()

#LLR=log(10^(-dat[,2]))

plot(dat[,1],-dat[,2],xlab="uv intensity",xlim=c(0,max(dat[,1])),ylim=c(min(-dat[,2]),max(-dat[,2],0)),ylab=expression('log'[10]~'(N/N'[0]~')'))

x = seq(from=0,to=max(dat[,1]),by=0.1)

par(lwd=2)

j=1; LL='data'

for (i in DesiredModels){

j=j+1

if (eval(parse(text=paste("length(formals(pred_",i,"))==3", sep='' ))) ){

eval(parse(text=paste("lines(x,pred_",i,"(output['",i,"','k1'],x,TRUE),col='",GSs[i,'color'],"',lty='",GSs[i,'lty'],"')" ,sep='')))

}else if(eval(parse(text=paste("length(formals(pred_",i,"))==4", sep='' ))) ){

eval(parse(text=paste("lines(x,pred_",i,"(output['",i,"','k1'],output['",i,"','k2'],x,TRUE),col='",GSs[i,'color'],"',lty='",GSs[i,'lty'],"')" ,sep='')))

}else if(eval(parse(text=paste("length(formals(pred_",i,"))==5", sep='' ))) ){

eval(parse(text=paste("lines(x,pred_",i,"(output['",i,"','k1'],output['",i,"','k2'],output['",i,"','k3'],x,TRUE),col='",GSs[i,'color'],"',lty='",GSs[i,'lty'],"')" ,sep='')))

}

#TODO: Build in autogenerated legend labels here.

LL[j] = eval(parse(text=paste("paste(GSs['",i,"','model'],': ',signif(output['",i,"','BIC'],3), sep='')",sep='' ))) #TODO: Fix.

}

legend('bottomleft',title='Model and BIC',legend=LL,lwd=c(1,rep(2,NROW(output))),lty=c(NA,as.character(GSs[DesiredModels,'lty'])),pch=c(1,rep(NA,NROW(output))),col=c('black',as.character(GSs[DesiredModels,'color'])),bg='transparent',fill='white',border='white') #TODO: Skip sapply() and simply con

title(main = datafile)

print('Final output:')

print(output)

#Writing output files: starting values, results, and the summary graph. MLE objects have already been output.

print(paste('This run saved files derived from the dataset ',dataset,' with the index ',OutputFileIndex,'.', sep=''))

write.csv(start,file=paste("Results/",dataset,"/start",dataset,OutputFileIndex,".csv",sep='')) #Writing start conditions to file.

write.csv(output,file=paste("Results/",dataset,"/output",dataset,OutputFileIndex,".csv",sep='')) #Writing output to file.

dev.print(png,filename=paste('Graphics/',dataset,'/Summary-',dataset,OutputFileIndex,'.png', sep=''),width=800,height=800)

#sfn = paste("Results/",dataset,"/start",dataset,".csv",sep='') #sfn: 'starting conditions file name'

#if (!file.exists(sfn)){

# write.csv(start,file=sfn) #Writing start conditions to file.

# write.csv(output,file=paste("Results/",dataset,"/output",dataset,".csv",sep='')) #Writing output to file.

# dev.print(png,filename=paste('Graphics/',dataset,'/Summary-',dataset,'.png', sep=''),width=800,height=800)

#} else {

# n=2

# while(file.exists(paste("Results/",dataset,"/start",dataset,n,".csv", sep=''))){

# n=n+1

# }

# write.csv(start,file=paste("Results/",dataset,"/start",dataset,n,".csv",sep='')) #Writing start conditions to file.

# write.csv(output,file=paste("Results/",dataset,"/output",dataset,n,".csv",sep='')) #Writing output to file.

# dev.print(png,filename=paste('Graphics/',dataset,'/Summary-',dataset,n,'.png', sep=''),width=800,height=800)

#}

#dev.print(png,filename=paste('Graphics/',dataset,'/Summary-',dataset,99,'.png', sep=''),width=500,height=500)

PersistModelLikFuncs.r

#Likelihood functions for use by other pieces of persistence model code.

#These functions call the prediction functions found in PersistModelPredFuncs.r.

#The gutsLX() functions at the beginning of this file are general aspects of 1, 2, & 3-parameter likelihood functions, regardless of the prediction function itself.

#The prediction function desired is an argument of the gutsLX() functions.

#Some likelihood functions have variants ending with ".TX". In these, a parameter is transformed before gutsLX() is called, to facilitate optimization.

gutsL1 = function(model,k1,sd,data){ #Common core of all 1-parameter likelihood functions for persistence models

t=data[,1]

LR=log(10^(-data[,2]))

lik_temp = matrix(nrow=NROW(LR),ncol=length(k1))

for (i in 1:length(k1)){

eval(parse(text=paste("lik_temp[,i] = dnorm(LR,log(pred_",model,"(k1[i],t,LRV=FALSE)),sd,log=TRUE)", sep='' )))

}

lik=-colSums(lik_temp)

lik

}

gutsL2 = function(model,k1,k2,sd,data){ #Common core of all 2-parameter likelihood functions for persistence models

t=data[,1]

LR=log(10^(-data[,2]))

ncols=1 #Default value corresponding to single values for k1 and k2; gets updated if multiple values for k1 or k2 are being used.

#if (length(k1) != length(k2)){stop('k1 and k2 are not the same length.')}

if (length(k1) > 1 | length(k2) > 1){

ks = expand.grid(list(k1=k1,k2=k2)) #TODO: apply()?

ncols = NROW(ks)

}else{ #if only single values for k1 and k2 are being used

ks = data.frame(k1=k1,k2=k2)

}

lik_temp = matrix(nrow=NROW(LR),ncol=ncols)

for (i in 1:ncols){

eval(parse(text=paste("lik_temp[,i]=dnorm(LR,log(pred_",model,"(ks[i,'k1'],ks[i,'k2'],t,LRV=FALSE)),sd,log=TRUE)", sep='' )))

}

lik=-colSums(lik_temp)

if (length(k1) > 1 | length(k2) > 1){

ks$nll = lik

lik = ks #If >1 set of parameters, output a dataset including parameter values & neg. log likelihood.

}

lik

}

gutsL3 = function(model,k1,k2,k3,sd,data){ #Common core of all 3-parameter likelihood functions for persistence models

t=data[,1]

LR=log(10^(-data[,2]))

ncols=1 #Default value corresponding to single values for k1 and k2; gets updated if multiple values for k1 or k2 are being used.

#if (length(k1) != length(k2)){stop('k1 and k2 are not the same length.')}

if (length(k1) > 1 | length(k2) > 1 | length(k3) > 1){

ks = expand.grid(list(k1=k1,k2=k2,k3=k3)) #TODO: apply()?

ncols = NROW(ks)

}else{ #if only single values for k1, k2, & k3 are being used

ks = data.frame(k1=k1,k2=k2,k3=k3)

}

lik_temp = matrix(nrow=NROW(LR),ncol=ncols)

for (i in 1:ncols){

eval(parse(text=paste("lik_temp[,i]=dnorm(LR,log(pred_",model,"(ks[i,'k1'],ks[i,'k2'],ks[i,'k3'],t,LRV=FALSE)),sd,log=TRUE)", sep='' )))

}

lik=-colSums(lik_temp)

if (length(k1) > 1 | length(k2) > 1 | length(k3) > 1){

ks$nll = lik

lik = ks #If >1 set of parameters, output a dataset including parameter values & neg. log likelihood.

}

lik

}

####exponential############

nll.ep = function(k1,sd,data){

gutsL1('ep',k1,sd,data)

}

####General logistic############ Misnomer - this is a 1-parameter logistic; the "generalized logistic" has ~4 parameters

nll.lg1 = function(k1,sd,data){

gutsL1('lg1',k1,sd,data)

}

####Fermi (2 param. logistic)############

nll.lg2 = function(k1,k2,sd,data){

gutsL2('lg2',k1,k2,sd,data)

}

nll.lg2.T1 = function(k1,k2,sd,data){

k1 = real2pos(k1,reverse=0)

k2 = real2pos(k2,reverse=0)

gutsL2('lg2',k1,k2,sd,data)

}

####exponential damped############

nll.epd = function(k1,k2,sd,data){

gutsL2('epd',k1,k2,sd,data)

}

####Juneja&Marks(1)############

nll.jm1 = function(k1,k2,sd,data){

gutsL2('jm1',k1,k2,sd,data)

}

nll.jm1.T1 = function(k1,k2,sd,data){

k1 = real2pos(k1,reverse=0)

k2 = real2pos(k2,reverse=0)

gutsL2('jm1',k1,k2,sd,data)

}

####Juneja&Marks(2)############

nll.jm2 = function(k1,k2,sd,data){

gutsL2('jm2',k1,k2,sd,data)

}

####Gompertz############

nll.gz = function(k1,k2,sd,data){

gutsL2('gz',k1,k2,sd,data)

}

nll.gz.T1 = function(k1,k2,sd,data){

k1 = real2extreme(k1,reverse=0)

k2 = real2extreme(k2,reverse=0)

gutsL2('gz',k1,k2,sd,data)

}

####Weibull############

nll.wb = function(k1,k2,sd,data){

gutsL2('wb',k1,k2,sd,data)

}

nll.wb.T1 = function(k1,k2,sd,data){

k1 = real2extreme(k1,reverse=0)

gutsL2('wb',k1,k2,sd,data)

}

####Lognormal############

nll.ln = function(k1,k2,sd,data){

gutsL2('ln',k1,k2,sd,data)

}

####Gamma############

nll.gam = function(k1,k2,sd,data){

gutsL2('gam',k1,k2,sd,data)

}

nll.gam.T1 = function(k1,k2,sd,data){

k1 = real2extreme(k1,reverse=0)

gutsL2('gam',k1,k2,sd,data)

}

####Biphasic spline############ This model is rather artificial, but it is useful for risk assessment.

nll.bi = function(k1,k2,sd,data){

gutsL2('bi',k1,k2,sd,data)

}

####Biphasic spline, 3 parameter############ This model is rather artificial, but it is useful for risk assessment.

nll.bi3 = function(k1,k2,k3,sd,data){

gutsL3('bi3',k1,k2,k3,sd,data)

}

nll.bi3.T1 = function(k1,k2,k3,sd,data){ #Variant with transformed k3

k3 = real2pos(k3,reverse=0)

gutsL3('bi3',k1,k2,k3,sd,data)

}

####Double exponential############

nll.dep = function(k1,k2,k3,sd,data){

gutsL3('dep',k1,k2,k3,sd,data)

}

nll.dep.T1 = function(k1,k2,k3,sd,data){ #Variant with transformed k3, assuming it's a probability

k3 = real2prob(k3,reverse=0)

gutsL3('dep',k1,k2,k3,sd,data)

}

nll.dep.T2 = function(k1,k2,k3,sd,data){ #Variant with transformed k3, assuming it's nonnegative.

k3 = real2extreme(k3,reverse=0)

gutsL3('dep',k1,k2,k3,sd,data)

}

####Gompertz 3############

nll.gz3 = function(k1,k2,k3,sd,data){

gutsL3('gz3',k1,k2,k3,sd,data)

}

nll.gz3.T1 = function(k1,k2,k3,sd,data){

k2 = real2pos(k2,reverse=0)

k3 = real2pos(k3,reverse=0)

gutsL3('gz3',k1,k2,k3,sd,data)

}

####Gompertz-Makeham############

nll.gzm = function(k1,k2,k3,sd,data){

gutsL3('gzm',k1,k2,k3,sd,data)

}

nll.gzm.T1 = function(k1,k2,k3,sd,data){

k1 = real2extreme(k1,reverse=0)

k2 = real2extreme(k2,reverse=0)

k3 = real2extreme(k3,reverse=0)

gutsL3('gzm',k1,k2,k3,sd,data)

}

####Sigmoid A (fast-slow-fast)############

nll.sA = function(k1,k2,k3,sd,data){

gutsL3('sA',k1,k2,k3,sd,data)

}

####Sigmoid B (slow-fast-slow)############

nll.sB = function(k1,k2,k3,sd,data){

gutsL3('sB',k1,k2,k3,sd,data)

}

PersistModelPredFuncs.r

#Prediction functions for use in other pieces of persistence model code.

#All of them output the proportion of microbes remaining; N(t)/N(0).

#Parameters for the functions are given by k1, k2, or k3; t is a vector of times.

#LRV=TRUE returns log10 reduction values, LRV=FALSE returns the proportion of microbes remaining.

#Some of these functions call other functions in ParamTransformations.r, if a parameter needs to be transformed to facilitate optimization.

####exponential############

pred_ep = function(k1,t,LRV){

#k1 = abs(k1)

out = exp(-k1*t)

if (LRV==TRUE) out=log10(out)

return(out)

}

####Biphasic spline, preset breakpoint############

pred_bi = function(k1,k2,t,LRV){ #k1 must be negative and k3 must be positive. k2 can be pos or neg. An increase in 2nd phase can happen if k2>k1.

warning("This biphasic spline model ('bi') is not recommended; it has a preset breakpoint of 72. Use 'bi3' instead.")

bp=72 #Breakpoint for the biphasic spline

out=rep(0,length(t));

if (min(t)<bp) out[t<bp] = exp(-k1*t[t<bp])

if (max(t)>=bp) out[t>=bp] = exp(-k1*t[t>=bp]+k2*(t[t>=bp]-bp))

if (LRV==TRUE) out=log10(out)

return(out)

}

####Biphasic spline, with breakpoint as parameter k3############

pred_bi3 = function(k1,k2,k3,t,LRV){ #k1 must be negative and k3 must be positive. k2 can be pos or neg. An increase in 2nd phase can happen if k2>k1. Reduces to exponential (with k1 as decay rate) if k3=0.

out=0;

if (min(t)<k3) out[t<k3] = exp(-k1*t[t<k3])

if (max(t)>=k3) out[t>=k3] = exp(-k1*t[t>=k3]+k2*(t[t>=k3]-k3))

if (LRV==TRUE) out=log10(out)

return(out)

}

####exponential damped############ Cavalli-Sforza 1983

pred_epd = function(k1,k2,t,LRV){

out = exp(-k1*t*exp(-k2*t)) #k1 must be pos. & k2 neg. to have a monotonic decline. If k1 and k2 are both pos. the curve eventually returns to the t0 level. Reduces to exponential if k2=0. If k1 is neg. then there is no decline from the initial level.

if (LRV==TRUE) out=log10(out)

return(out)

}

####Juneja&Marks(1)############

pred_jm1 = function(k1,k2,t,LRV){ #k1 increases speed of decline. k2>1 gives shouldering & k2<1 gives tailing. Reduces to exponential if k2=1.

#k1=abs(k1)

#k2=abs(k2)

out = 1-(1-exp(-k1*t))^k2

if (LRV==TRUE) out=log10(out)

return(out)

}

####Juneja&Marks(2)############

pred_jm2 = function(k1,k2,t,LRV){ #Always tails. Neg. k1 is acceptable. Decreasing k2 leads to more rapid plateauing. Juneja VK 200, eq. 1?

#k2=abs(k2)

out = 1/(1+exp(k1+k2*log(t)))

if (LRV==TRUE) out=log10(out)

return(out)

}

####General logistic############ See Kamau DN 1990. The name is a misnomer (generalized logistic has 4 parameters). TODO: 2-param. logistic, maybe 3-param.

pred_lg1 = function(k1,t,LRV){ #Slower decay than exponential for same value of k1; weak shouldering.

#log(2)-log(1+exp(-k1*t)) #log(2) forces time of 0 to output 1

#k1 = abs(k1)

out = 2*1/(1+exp(k1*t)) #2 at beginning forces time of 0 to output 1. NOTE: check graphically if this is a simplified form of JM2.

if (LRV==TRUE) out=log10(out)

return(out)

}

####2 parameter logistic############ Also known as a Fermi curve. See p.24, Peleg 2006, Advanced Quantitative Microbiology for Foods and Biosystems, CRC Press.

pred_lg2 = function(k1,k2,t,LRV){ #Slower decay than exponential for same value of k1; weak shouldering.

#Only works if k2 >> k1; otherwise, Nt/N0 can be very different from 1 at time 0.

out = 1/(1+exp(k1*(t-k2))) #2 at beginning forces time of 0 to output 1. k2 is the time at the inflection point, and k1 is the decay rate at the inflection point.

if (LRV==TRUE) out=log10(out)

return(out) #Only starts at 1 (or 0 on log scale) if k1 and k2 are both 'large', meaning that the product of k1 and k2 should be > ~7.

}

####Gompertz############

pred_gz = function(k1,k2,t,LRV){ #Gives shouldering if k1 & k2 are both +, or cessation of decay at y=exp(-k1) if k1 & k2 are both -. Increasing k1 or k2 increases the rate of decline.

#if (sign(k1) != sign(k2)) stop('k1 and k2 must have the same sign: both + for shouldering, both - for tailing.')

#out = exp(-k1*exp(k2*t)+k1) #k1 at end forces time of 0 to output 1.

#out = exp(-k1*(exp(k2*t)-1)) #From Wikipedia; 1 minus the cumulative form of the Gompertz distribution.

out = exp(-k1/k2*(exp(k2*t)-1)) #Differs from Wikipedia; see Wu JW 2004, eq. 2. Also El-Gohary A 2013, eq. 1 with theta=1. Also 'gzm' with k3=0.

if (LRV==TRUE) out=log10(out)

return(out)

}

####Gompertz (3 parameter) ############ Gil 2011, eq. 14 (see also eq. 7). k1 is 'tail', k2 is max. inact. rate, k3 is 'shoulder'.

pred_gz3 = function(k1,k2,k3,t,LRV){ #Gives shouldering if k1 & k2 are both +, or cessation of decay at y=exp(-k1) if k1 & k2 are both -. Increasing k1 or k2 increases the rate of decline.

#out = k3*exp(-exp(k2*(log(t)-k1))) #Variant from spreadsheet; not sure of its source.

#out = exp(-k3*t - k1/k2*(exp(k2*t)-1)) #Gompertz-Makeham CDF from Wikipedia.

out = 10^(k1*exp(-exp((-k2*exp(1)*(k3-t)/k1)+1))) #Gil 2011. k1 is 'tail', k2 is max. inact. rate, k3 is 'shoulder'. Might be a convenient reparameterization, but != 0 at t=0.

if (LRV==TRUE) out=log10(out)

return(out)

}

####Gompertz-Makeham ############

pred_gzm = function(k1,k2,k3,t,LRV){ #k3 must be positive for decay.

out = exp(-k3*t - k1/k2*(exp(k2*t)-1)) #Gompertz-Makeham CDF from Wikipedia. If k3 = 0, we get 2-parameter Gompertz.

if (LRV==TRUE) out=log10(out)

return(out)

}

####Weibull############ Coroller 2006. Other sources (e.g., Wikipedia) use e as the base instead of 10, but using 10 means that k1 conveniently equals the T90.

pred_wb = function(k1,k2,t,LRV){ #k1 is the time needed for the 1st log10 reduction (delta); k2 is a shape parameter (P). Both must be positive.

out = 10^(-((t/k1)^k2)) #If k2 < 1, there is tailing; if k2 > 1, there is shouldering.

if (LRV==TRUE) out=log10(out) #Equivalent to exponential model if k2==1. k1 for the equivalent exp. model then equals -log(10^(-1/k1)).

return(out)

}

####Double exponential############ See p.6, Peleg 2006, Advanced Quantitative Microbiology for Foods and Biosystems, CRC Press. See also GInaFiT.

pred_dep = function(k1,k2,k3,t,LRV){ #k1 and k2 are decay rates for each of 2 microbe populations. k3 is the proportion of the total population subject to k1.

out = k3 * exp(-k1*t) + (1-k3) * exp(-k2*t) #But see Abraham 1990: allowing k3 to go above 1 gives 'activation shoulder' behavior.

if (LRV==TRUE) out=log10(out) #Equivalent to exponential model if k2==1. k1 for the equivalent exp. model then equals -log(10^(-1/k1)).

return(out)

}

####Gamma####

pred_gam = function(k1,k2,t,LRV){

out = 1-pgamma(t,shape=k2,scale=k1) #If shape=1 (k2=1), reduces to exponential.

if (LRV==TRUE) out=log10(out)

return(out)

}

####Lognormal#### Aragao 2007. Behaves similarly to the Weibull model, but becomes loglinear sooner if there is shouldering.

pred_ln = function(k1,k2,t,LRV){

out = 1-plnorm(t,meanlog=k1,sdlog=k2)

if (LRV==TRUE) out=log10(out)

return(out)

}

####Sigmoid A (fast-slow-fast)#### See p.35, Peleg 2006, Advanced Quantitative Microbiology for Foods and Biosystems, CRC Press.

pred_sA = function(k1,k2,k3,t,LRV){

out = 10^-(k1*t/((1+k2*t)*(k3-t)))

if (LRV==TRUE) out=log10(out)

return(out)

}

####Sigmoid B (slow-fast-slow)#### See p.36, Peleg 2006, Advanced Quantitative Microbiology for Foods and Biosystems, CRC Press.

pred_sB = function(k1,k2,k3,t,LRV){

out = 10^-(k1*t^k3/(k2+t^k3)) #k1 appears to be the max LRV.

if (LRV==TRUE) out=log10(out)

return(out)

}

LikPlot1.r

#Generates likelihood plot after a one-parameter persistence model has been fit, as a check.

#model: The abbreviation of the model used.

#k1lim: Multipliers of k parameters that are desired (2-item vectors).

#k1sc: Whether to use log scale (*) or linear scale (+) for the k parameters that are tried

#output: The dataset resulting from running various persistence model fits.

LikPlot1 = function(model,k1lim,k1sc,output,dataname){

LO = 1000

png(paste('Graphics/',dataname,'/TestLikPlot-',dataname,'-',model,'.png',sep=''))

if (k1sc=='+'){

k1Try = seq(from=output[model,'k1']+k1lim[1],to=output[model,'k1']+k1lim[2], length.out=LO)

XLAB = 'k1'

}else if (k1sc=='*'){

k1Try = sign(output[model,'k1']) * 10^seq(from=log10(abs(output[model,'k1'])*k1lim[1]),to=log10(abs(output[model,'k1'])*k1lim[2]), length.out=LO)

if (sign(output[model,'k1'])==1){XLAB = 'log10(k1)'} else if (sign(output[model,'k1'])==-1){XLAB = 'log10(|k1|)'}

}

eval(parse(text=paste("nll = nll.",model,"(k1Try,output['",model,"','sd'],dat)", sep='' )))

nll = data.frame(k1=k1Try,nll=nll)

print('Summary of all parameters tried by brute force:')

print(summary(nll))

print('Apparent best fit by brute force:')

gMLEs = nll[which(nll$nll==min(nll$nll)),]

print(head(gMLEs)) #Sometimes gMLEs can have >1 row; not sure why.

print(paste("Attempting to estimate the sd parameter based on brute force results for",model,"..."))

sdStart = list(sd=output[model,'sd'])

eval(parse(text=paste("sdResults = mle(nll.",model,", start=sdStart, method='Brent', lower=0, upper=10*output[model,'sd'], control=controllist, fixed=list(k1=",gMLEs[,'k1'],",data=dat))", sep='')))

print('Estimate of sd parameter, based on preexisting brute force estimate of other parameters:')

gMLEsd = coef(sdResults)[c('k1','sd')]

print(gMLEsd)

if (min(gMLEs[,'k1'])==min(k1Try) | max(gMLEs[,'k1'])==max(k1Try)){

warning(paste('Brute force MLE for',model,'is on the border of the range of values tried. Expand the range.'))

}

if (k1sc=='+'){

plot(nll$k1,nll$nll,main=paste('Likelihood plot, ',dataname,', model ',model, sep=''), xlab=XLAB, ylab='-log(Likelihood)')

}else if (k1sc=='*'){ #NOTE: May throw an error here if there's ever a 1-parameter model with a negative parameter.

plot(nll$k1,nll$nll,log='x',main=paste('Likelihood plot, ',dataname,', model ',model, sep=''), xlab=XLAB, ylab='-log(Likelihood)')

}

abline(h=gMLEs[1,'nll'],col='red',lwd=3) #Marks the minimum neg. log likelihood for all values of k1 tried.

abline(v=gMLEs[,'k1'],col='red',lwd=3)

abline(v=output[model,'k1']) #Hopefully the MLE from mle() overlaps the minimum.

abline(h=output[model,'nll'])

legend('topleft',legend=c('Optimized MLE','Brute force MLE'),lty='solid',col=c('black','red'),lwd=c(1,3))

dev.off()

eval(parse(text=paste("nll_sd = nll.",model,"(gMLEsd['k1'],gMLEsd['sd'],dat)", sep="")))

print('Revised neg. log likelihood based on new brute force sd:')

print(nll_sd)

gMLEs[1,'nll'] = nll_sd

gMLEs[1,'sd'] = gMLEsd['sd']

r = output[model,'k1'] / gMLEs[1,'k1'] #Ratio of optimized MLE to brute force MLE.

if (r > 1.1 | r < 0.9){warning('Optimized and brute force MLEs for k1 differ by >10%. Use brute force MLE as a guide to pick different starting values.')}

return(gMLEs)

}

LikPlot2.r

#Generates contour likelihood plots after a 2-parameter persistence model has been fit, as a check.

#Essentially trusts mle() to get the signs of the parameters right.

#model: The abbreviation of the model used.

#k1lim & k2lim: Define ranges of k parameters that are desired (2-item vectors).

#k1sc, k2sc: Whether to use log scale (*) or linear scale (+) for the k parameters that are tried

#output: The dataset resulting from running various persistence model fits.

LikPlot2 = function(model,k1lim,k1sc,k2lim,k2sc,output,dataname){

LO = 100 #Value of length.out argument for all seq() calls

png(paste('Graphics/',dataname,'/TestLikContour-',dataname,'-',model,'.png',sep=''))

#Generating trial values for the two parameters.

if (k1sc == '+'){

k1Try = seq(from=output[model,'k1']+k1lim[1], to=output[model,'k1']+k1lim[2], length.out=LO)

XLAB = 'k1'

}else if (k1sc == '*'){

k1Try = sign(output[model,'k1']) * 10^seq(from=log10(abs(output[model,'k1'])*k1lim[1]), to=log10(abs(output[model,'k1'])*k1lim[2]), length.out=LO)

if (sign(output[model,'k1'])==1){XLAB = 'log10(k1)'} else if (sign(output[model,'k1'])==-1){XLAB = 'log10(|k1|)'}

}

if (k2sc == '+'){

k2Try = seq(from=output[model,'k2']+k2lim[1], to=output[model,'k2']+k2lim[2], length.out=LO)

YLAB = 'k2'

}else if (k2sc == '*'){

k2Try = sign(output[model,'k2']) * 10^seq(from=log10(abs(output[model,'k2'])*k2lim[1]), to=log10(abs(output[model,'k2'])*k2lim[2]), length.out=LO)

if (sign(output[model,'k2'])==1){YLAB = 'log10(k2)'} else if (sign(output[model,'k2'])==-1){YLAB = 'log10(|k2|)'}

}

eval(parse(text=paste("nll = nll.",model,"(k1Try,k2Try,output[model,'sd'],dat)", sep=""))) #Calculating prelim. likelihoods for all possible param. combos.

print('Summary of all parameters tried by brute force:')

print(summary(nll))

#print(paste('nllMin=',min(nll),', nllMax=',max(nll),sep=''))

print('Apparent best fit by brute force:')

gMLEs = nll[which(nll$nll==min(nll$nll)),]

print(head(gMLEs)) #Sometimes gMLEs can have >1 row; not sure why.

#TODO: Further check this code block, and apply to LikPlot1.r & LikPlot3.r.

print(paste("Attempting to estimate the sd parameter based on brute force results for",model,"..."))

sdStart = list(sd=output[model,'sd'])

eval(parse(text=paste("sdResults = mle(nll.",model,", start=sdStart, method='Brent', lower=0, upper=10*output[model,'sd'], control=controllist, fixed=list(k1=",gMLEs[,'k1'],",k2=",gMLEs[,'k2'],",data=dat))", sep='')))

print('Estimate of sd parameter, based on preexisting brute force estimate of other parameters:')

gMLEsd = coef(sdResults)[c('k1','k2','sd')]

print(gMLEsd)

#TODO: Recalculate likelihood based on the new sd estimate!

if (min(gMLEs[,'k1'])==min(k1Try) | max(gMLEs[,'k1'])==max(k1Try)){

warning(paste('Brute force MLE for k1 of',model,'is on the border of the range of values tried. Expand the range.'))

}

if (min(gMLEs[,'k2'])==min(k2Try) | max(gMLEs[,'k2'])==max(k2Try)){

warning(paste('Brute force MLE for k2 of',model,'is on the border of the range of values tried. Expand the range.'))

}

#Now making the chart. Will be log-log, semilog, or arithmetic, as appropriate.

MAIN = paste('Neg. log likelihood for differing k1 & k2,',dataname,', model',model)

if (k1sc=='+'){

if (k2sc=='+'){

print(levelplot(nll ~ k1 + k2,data=nll,xlab=XLAB,ylab=YLAB,main=MAIN,col.regions=terrain.colors(100)))

trellis.focus("panel", 1, 1, highlight=FALSE) #Don't understand this, but it's necessary.

lpoints(x=output[model,'k1'],y=output[model,'k2'],pch=1,col='black')

lpoints(x=gMLEs[,'k1'],y=gMLEs[,'k2'],pch=3,col='black')

print(paste("X",k1sc,"by Y",k2sc))

}else if (k2sc=='*'){

print(levelplot(nll ~ k1 + log10(abs(k2)),data=nll,xlab=XLAB,ylab=YLAB,main=MAIN,col.regions=terrain.colors(100)))

trellis.focus("panel", 1, 1, highlight=FALSE) #Don't understand this, but it's necessary.

lpoints(x=output[model,'k1'],y=log10(abs(output[model,'k2'])),pch=1,col='black')

lpoints(x=gMLEs[,'k1'],y=log10(abs(gMLEs[,'k2'])),pch=3,col='black')

print(paste("X",k1sc,"by Y",k2sc))

}

}else if (k1sc=='*'){

if (k2sc=='+'){

print(levelplot(nll ~ log10(abs(k1)) + k2,data=nll,xlab=XLAB,ylab=YLAB,main=MAIN,col.regions=terrain.colors(100)))

trellis.focus("panel", 1, 1, highlight=FALSE) #Don't understand this, but it's necessary.

lpoints(x=log10(abs(output[model,'k1'])),y=output[model,'k2'],pch=1,col='black')

lpoints(x=log10(abs(gMLEs[,'k1'])),y=gMLEs[,'k2'],pch=3,col='black')

print(paste("X",k1sc,"by Y",k2sc))

}else if (k2sc=='*'){

print(levelplot(nll ~ log10(abs(k1)) + log10(abs(k2)),data=nll,xlab=XLAB,ylab=YLAB,main=MAIN,col.regions=terrain.colors(100)))

trellis.focus("panel", 1, 1, highlight=FALSE) #Don't understand this, but it's necessary.

lpoints(x=log10(abs(output[model,'k1'])),y=log10(abs(output[model,'k2'])),pch=1,col='black')

lpoints(x=log10(abs(gMLEs[,'k1'])),y=log10(abs(gMLEs[,'k2'])),pch=3,col='black')

print(paste("X",k1sc,"by Y",k2sc))

}

}

dev.off()

eval(parse(text=paste("nll_sd = nll.",model,"(gMLEsd['k1'],gMLEsd['k2'],gMLEsd['sd'],dat)", sep="")))

print('Revised neg. log likelihood based on new brute force sd:')

print(nll_sd)

gMLEs[1,'nll'] = nll_sd

gMLEs[1,'sd'] = gMLEsd['sd']

r = output[model,c('k1','k2')] / gMLEs[1,c('k1','k2')] #Ratio of optimized MLE to 1st brute force MLE.

if (max(r) > 1.1 | min(r) < 0.9){warning('Optimized and brute force MLEs for one or both parameters differ by >10%. Use brute force MLE as a guide to pick different starting values.')}

return(gMLEs) #Returns MLEs based on graph, for reuse as initial estimates if necessary.

}

LikPlot3.r

#Generates contour likelihood plots after a 3-parameter persistence model has been fit, as a check.

#Essentially trusts mle() to get the signs of the parameters right.

#model: The abbreviation of the model used.

#k1lim, k2lim, k3lim: Define ranges of k parameters that are desired (2-item vectors).

#k1sc, k2sc: Whether to use log scale (*) or linear scale (+) for the k parameters that are tried

#The intervals for k1lim and k2lim are logarithmic (or sometimes arithmetic), but for k3lim, they are always arithmetic. k3lim defines each of the subpanels on the plot, so it is the most granular.

#output: The dataset resulting from running various persistence model fits.

LikPlot3 = function(model,k1lim,k1sc,k2lim,k2sc,k3lim,output,dataname){

LO = 50 #Note: Only applies to k1 and k2, not k3.

print(paste('Attempting to create multipanel contour plot for the',model,'model, dataset',dataname,'.'))

if (k1sc=='+'){

k1Try = seq(from=output[model,'k1']+k1lim[1], to=output[model,'k1']+k1lim[2], length.out=LO)

XLAB = 'k1'

}else if (k1sc=='*'){

k1Try = sign(output[model,'k1']) * 10^seq(from=log10(abs(output[model,'k1'])*k1lim[1]), to=log10(abs(output[model,'k1'])*k1lim[2]), length.out=LO)

if (sign(output[model,'k1'])==1){XLAB = 'log10(k1)'} else if (sign(output[model,'k1'])==-1){XLAB = 'log10(|k1|)'}

}

if (k2sc=='+'){

k2Try = seq(from=output[model,'k2']+k2lim[1], to=output[model,'k2']+k2lim[2], length.out=LO)

YLAB = 'k2'

}else if (k2sc=='*'){

k2Try = sign(output[model,'k2']) * 10^seq(from=log10(abs(output[model,'k2'])*k2lim[1]), to=log10(abs(output[model,'k2'])*k2lim[2]), length.out=LO)

if (sign(output[model,'k2'])==1){YLAB = 'log10(k2)'} else if (sign(output[model,'k2'])==-1){YLAB = 'log10(|k2|)'}

}

ZLAB = 'k3'

k3Try = unique(sort(c(seq(from=k3lim[1], to=k3lim[2], length.out=11), output[model,'k3']))) #Need unique() because sometimes the MLE of k3 and a 'tried' value are identical.

print(paste('Trying these values for k3:',paste(format(k3Try,digits=2),collapse=' ') ))

print(paste('k1min=',min(k1Try),', k1max=',max(k1Try),', k2min=',min(k2Try),', k2max=',max(k2Try),', k3min=',min(k3Try),', k3max=',max(k3Try),sep=''))

eval(parse(text=paste("nll = nll.",model,"(k1Try,k2Try,k3Try,output[model,'sd'],dat)", sep=""))) #Getting neg. log likelihoods for all parameter combinations.

print('Summary of all parameters tried by brute force:')

print(summary(nll))

print('Apparent best fit by brute force:')

gMLEs = nll[which(nll$nll==min(nll$nll[!is.na(nll$nll)])),]

print(head(gMLEs)) #Sometimes gMLEs can have >1 row, for example if a 3-param. model is reduced to a 2-param.

print(paste("Attempting to estimate the sd parameter based on brute force results for",model,"..."))

sdStart = list(sd=output[model,'sd'])

eval(parse(text=paste("sdResults = mle(nll.",model,", start=sdStart, method='Brent', lower=0, upper=10*output[model,'sd'], control=controllist, fixed=list(k1=",gMLEs[1,'k1'],",k2=",gMLEs[1,'k2'],",k3=",gMLEs[,'k3'],",data=dat))", sep='')))

print('Estimate of sd parameter, based on preexisting brute force estimate of other parameters:')

gMLEsd = coef(sdResults)[c('k1','k2','k3','sd')]

print(gMLEsd)

if (min(gMLEs[,'k1'])==min(k1Try) | max(gMLEs[,'k1'])==max(k1Try)){

warning(paste('Brute force MLE for k1 of ',model,' is on the border of the range of values tried. Expand the range.'))

}

if (min(gMLEs[,'k2'])==min(k2Try) | max(gMLEs[,'k2'])==max(k2Try)){

warning('Brute force MLE for k2 of ',model,' is on the border of the range of values tried. Expand the range.')

}

if (min(gMLEs[,'k3'])==min(k3Try) | max(gMLEs[,'k3'])==max(k3Try)){

warning('Brute force MLE for k3 of ',model,' is on the border of the range of values tried. Expand the range.')

}

nll$k3 = factor(nll$k3) #Needs to be a factor in order to divide up the levelplots by levels of k3.

#Now making the charts. Will be log-log, semilog, or arithmetic, depending on k1sc and k2sc.

trellis.device(device='png',file=paste('Graphics/',dataname,'/TestLikMultiContour-',dataname,'-',model,'.png',sep=''),height=750,width=800)

MAIN = paste('Neg. log likelihood for differing k1, k2, & k3,',dataname,', model',model)

mleIndex = which(k3Try == output[model,'k3']) #The index of the panel that contains the actual k3 value ouputted by the mle() optimization.

print(paste('Column & row for the MLE:',mleIndex))

graphIndex = which(k3Try == nll[which(nll$nll==min(nll$nll[!is.na(nll$nll)]))[1],'k3']) #The index(es) of the panel(s) containing the brute-force MLE(s).

mlePanel = c(mleIndex%%4,ceiling(mleIndex/4)) #There can be only 1 MLE from mle().

if (mlePanel[1]==0) mlePanel[1]=4 #A positive nonzero number is required for determining panel column.

print(mlePanel)

graphPanelCol = graphIndex%%4 #There can be multiple MLEs by brute force, so must handle columns and rows for the panels separately.

graphPanelRow = ceiling(graphIndex/4)

graphPanelCol[graphPanelCol==0] = 4 #A positive nonzero number is required for determining panel column.

print(graphPanelCol); print(graphPanelRow)

if (k1sc=='+'){

if (k2sc=='+'){

k1k2 = levelplot(nll ~ k1 * k2 | k3, data=nll,xlab=XLAB,ylab=YLAB,main=MAIN,col.regions=terrain.colors(100))

print(k1k2) #This actually sends the new levelplot to the graphics device.

trellis.focus("panel", mlePanel[1], mlePanel[2])

lpoints(x=output[model,'k1'],y=output[model,'k2'],pch=1,col='black')

trellis.unfocus()

for (i in 1:length(graphPanelCol)){

if (i==2){warning('Brute force has returned multiple MLEs!')}

trellis.focus("panel", graphPanelCol[i], graphPanelRow[i])

lpoints(x=gMLEs[i,'k1'],y=gMLEs[i,'k2'],pch=3,col='black')

trellis.unfocus()

}

}else if (k2sc=='*'){

k1k2 = levelplot(nll ~ k1 * log10(abs(k2)) | k3, data=nll,xlab=XLAB,ylab=YLAB,main=MAIN,col.regions=terrain.colors(100))

print(k1k2) #This actually sends the new levelplot to the graphics device.

trellis.focus("panel", mlePanel[1], mlePanel[2])

lpoints(x=output[model,'k1'],y=log10(abs(output[model,'k2'])),pch=1,col='black')

trellis.unfocus()

for (i in 1:length(graphPanelCol)){

if (i==2){warning('Brute force has returned multiple MLEs!')}

trellis.focus("panel", graphPanelCol[i], graphPanelRow[i])

lpoints(x=gMLEs[i,'k1'],y=log10(abs(gMLEs[i,'k2'])),pch=3,col='black')

trellis.unfocus()

}

}

} else if (k1sc=='*'){

if (k2sc=='+'){

k1k2 = levelplot(nll ~ log10(abs(k1)) * k2 | k3, data=nll,xlab=XLAB,ylab=YLAB,main=MAIN,col.regions=terrain.colors(100))

print(k1k2) #This actually sends the new levelplot to the graphics device.

trellis.focus("panel", mlePanel[1], mlePanel[2])

lpoints(x=log10(abs(output[model,'k1'])),y=output[model,'k2'],pch=1,col='black')

trellis.unfocus()

for (i in 1:length(graphPanelCol)){

if (i==2){warning('Brute force has returned multiple MLEs!')}

trellis.focus("panel", graphPanelCol[i], graphPanelRow[i])

lpoints(x=log10(abs(gMLEs[i,'k1'])),y=gMLEs[i,'k2'],pch=3,col='black')

trellis.unfocus()

}

}else if (k2sc=='*'){

k1k2 = levelplot(nll ~ log10(abs(k1)) * log10(abs(k2)) | k3, data=nll,xlab=XLAB,ylab=YLAB,main=MAIN,col.regions=terrain.colors(100))

print(k1k2) #This actually sends the new levelplot to the graphics device.

trellis.focus("panel", mlePanel[1], mlePanel[2])

lpoints(x=log10(abs(output[model,'k1'])),y=log10(abs(output[model,'k2'])),pch=1,col='black')

trellis.unfocus()

for (i in 1:length(graphPanelCol)){

if (i==2){warning('Brute force has returned multiple MLEs!')}

trellis.focus("panel", graphPanelCol[i], graphPanelRow[i])

lpoints(x=log10(abs(gMLEs[i,'k1'])),y=log10(abs(gMLEs[i,'k2'])),pch=3,col='black')

trellis.unfocus()

}

}

}

dev.off()

eval(parse(text=paste("nll_sd = nll.",model,"(gMLEsd['k1'],gMLEsd['k2'],gMLEsd['k3'],gMLEsd['sd'],dat)", sep="")))

print('Revised neg. log likelihood based on new brute force sd:')

print(nll_sd)

gMLEs[1,'nll'] = nll_sd

gMLEs[1,'sd'] = gMLEsd['sd']

r = (output[model,c('k1','k2','k3')]+1e-6) / (gMLEs[1,c('k1','k2','k3')]+1e-6) #Ratio of optimized MLE to brute force MLE. Adding a small amount to avoid errors from 0/0.

print(r)

if (max(r) > 1.1 | min(r) < 0.9){warning('Optimized and brute force MLEs for one or more parameters differ by >10%. Use brute force MLE as a guide to pick different starting values.')}

return(gMLEs[1,]) #Returns MLEs based on graph, for reuse as initial estimates if necessary.

}

ParamTransformations.r

#Useful functions for transforming parameters.

#Some models have constraints on the domains of their parameters. However, these aren't tolerated well by mle() or optim().

#Therefore, these functions are used to transform nonnegative numbers, proportions, huge/tiny values, etc. to something more manageable by mle().

#In the function names, 'real' means a number on the real number line that is suitable for optimization:

# i.e., pos, neg, or 0, and neither huge nor tiny in magnitude.

#nthroot function grabbed from http://rosettacode.org/wiki/Nth_root#R

nthroot <- function(A, n, tol=sqrt(.Machine$double.eps))

{

ifelse(A < 1, x0 <- A * n, x0 <- A / n)

repeat

{

x1 <- ((n-1)*x0 + A / x0^(n-1))/n

if(abs(x1 - x0) > tol) x0 <- x1 else break

}

x1

} #Returns the single real root if n is odd. Does not work if A is negative and n is even, because then no real root exists.

real2prob = function(x,reverse){ #Logistic function: convert real numbers to probability scale [0,1].

if (reverse==1){

y=-log(1/x-1)

}else{

y=1/(1+exp(-x))

}

return(y)

}

real2pos = function(x,reverse){ #Seems obvious, but implementing it this way for consistency.

if (reverse==1){

y=log(x)

}else{

y=exp(x)

}

return(y)

}

real2extreme = function(x,reverse){ #In reverse, makes extremely huge- or tiny-magnitude numbers closer to 1, preserving the sign.

if (reverse==1){

y = nthroot(x,7) #Real 7th root. Could change 7 to some other odd integer if desired.

}else{

y = x^7

}

}

Transform.r

#Transforming all output variables at once (i.e., from real numbers to a smaller set).

#Transforms 'start' or 'output' dataset, using 'GSs' dataset from PersistFitKSExx.r.

#REVERSE is 1 for transformation to real numbers, 0 for back-transformation from real numbers.

#Based on the contents of 'GSs', it will also call some functions from ParamTransformations.r.

Transform = function(data,GSs,REVERSE){

if (REVERSE==1){

cat('Transforming to real: ')

}else if (REVERSE==0){

cat('Transforming from real: ')

}

for (i in rownames(data)){ #Actually transforming the starting variables to real number scale using GSs

for (j in c('k1','k2','k3','k4','k5')){

if (GSs[i,j] != '-'){

#if (REVERSE==1 || grepl('kSource',paste(names(data),collapse=''))==FALSE || data[i,'kSource'] != 'BF'){

cat(paste(j,'for',i,'using',GSs[i,j],'; '))

data[i,j] = eval(parse(text=paste(GSs[i,j],"(",data[i,j],",reverse=",REVERSE,")", sep='')))

#}

}

}

}

return(data)

}
